# Supplementary material for: Master Regulators and Cofactors of Human Neuronal Cell Fate Specification Identified by CRISPR Gene Activation Screens
Source: Cell Rep. Author manuscript; Available in PMC 2020 Dec 11. (PMC7730023; doi:10.1016/j.celrep.2020.108460)
Supplement: 1 [file NIHMS1651252-supplement-1.pdf]

Cell Reports, Volume *33*

Supplemental Information

Master Regulators and Cofactors of Human Neuronal Cell Fate Specification Identified by CRISPR Gene Activation Screens

Joshua B. Black, Sean R. McCutcheon, Shataakshi Dube, Alejandro Barrera, Tyler S. Klann, Grayson A. Rice, Shaunak S. Adkar, Scott H. Soderling, Timothy E. Reddy, and Charles A. Gersbach

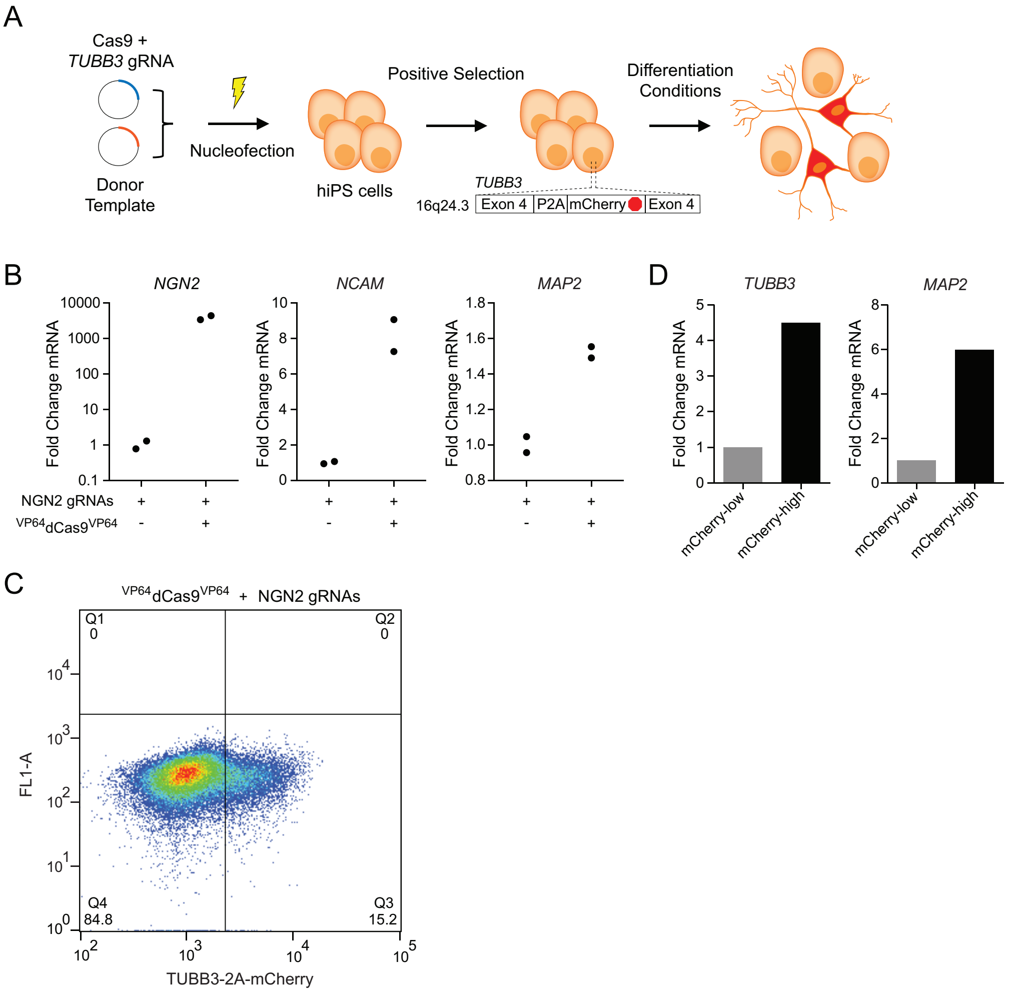

Figure S1. Generation and characterization of a *TUBB3-2A-mCherry* reporter cell line. (A) Schematic representation of the knock-in of a *P2A-mCherry* cassette into exon four of *TUBB3* in a human pluripotent stem cell line using Cas9 nuclease and a donor template. (B) Targeted activation of endogenous *NEUROG2* in pluripotent stem cells with ^{VP64}dCas9^{VP64} and a set of four gRNAs targeting the *NEUROG2* promoter. Expression of *NCAM* (middle) and *MAP2* (right) with targeted activation of *NEUROG2* (n = 2 biological replicates). (C) TUBB3-2A-mCherry expression by flow cytometry with targeted activation of *NEUROG2* with ^{VP64}dCas9^{VP64} and a set of four gRNAs targeting the promoter. (D) *TUBB3* and *MAP2* expression in *TUBB3-2A-mCherry* cells sorted for the highest and lowest mCherry expression after activation of *NEUROG2* with ^{VP64}dCas9^{VP64} and gRNAs (n = 1 biological replicate). Related to Figure 1.

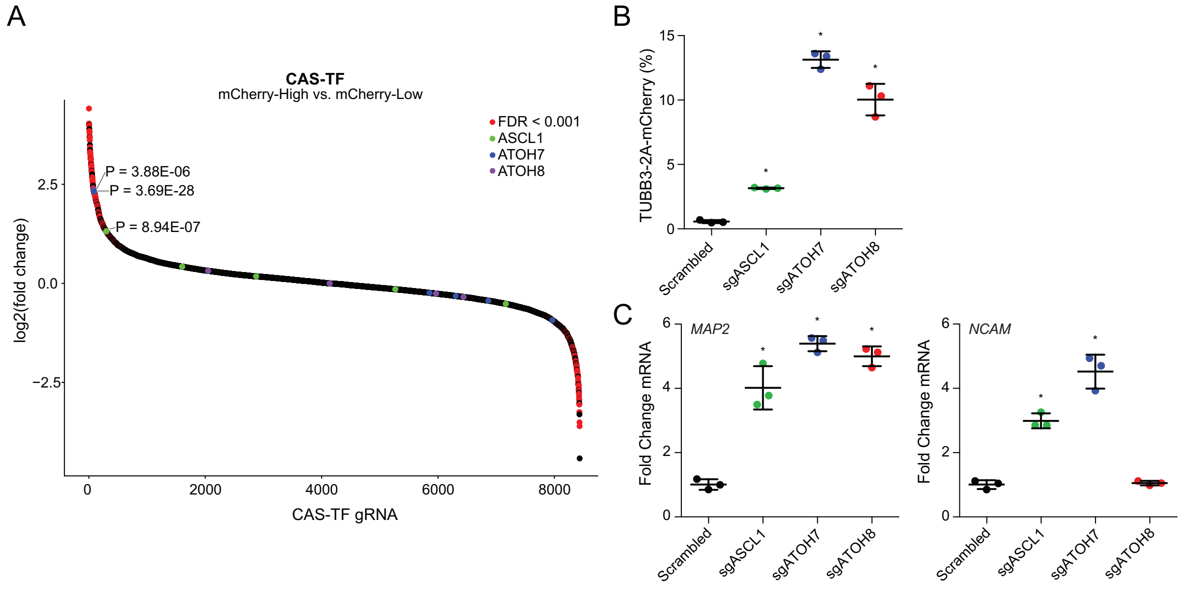

Figure S2. Validations of TFs with a single enriched gRNA. (A) A ranked list of fold change in gRNA abundance between mCherry-High versus mCherry-Low expressing cells in the single factor CAS-TF screen. *ASCL1*, *ATOH7* and *ATOH8* all have a single gRNA significantly enriched. (B) Individual validations of sgASCL1, sgATOH7 and sgATOH8 for (B) percent TUBB3-2A-mCherry expression and (C) *MAP2* (left) and *NCAM* (right) expression four days after gRNA transduction (*p < 0.05 by global one-way ANOVA with Dunnett's post hoc test comparing all groups to a scrambled gRNA, n = 3 biological replicates, error bars represent SEM). Related to Figures 1 and 2.

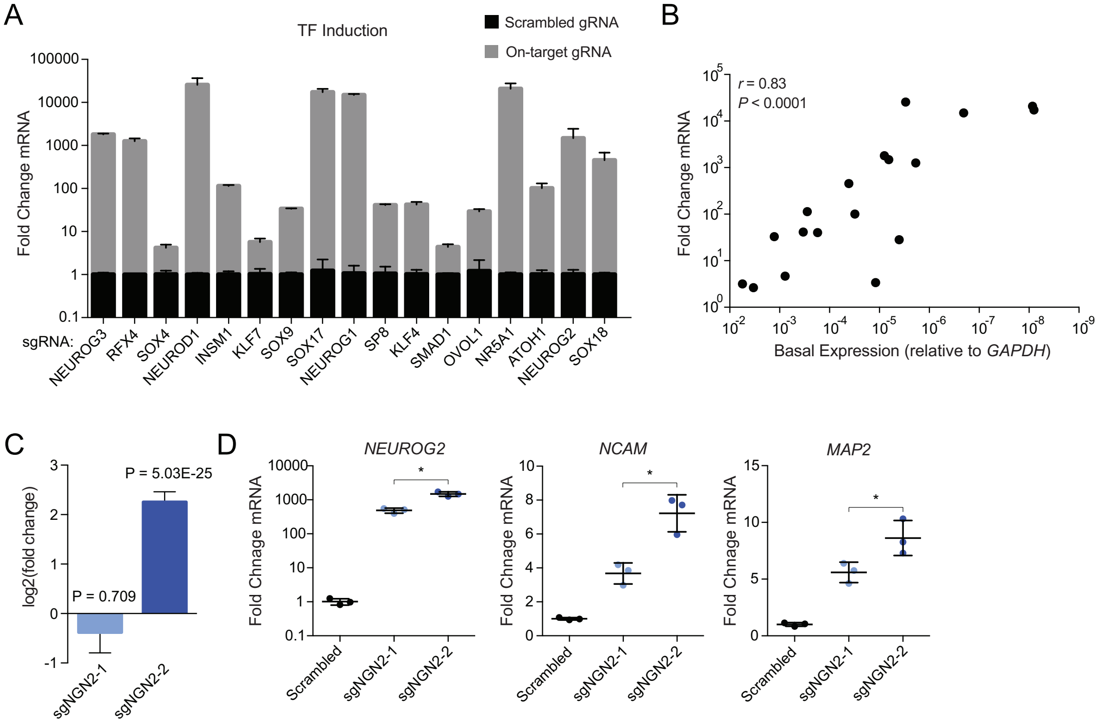

Figure S3. Endogenous induction of TFs with ^{VP64}dCas9^{VP64}. (A) Fold induction of a subset of 17 TFs enriched in the single factor CAS-TF screen with ^{VP64}dCas9^{VP64} and the top enriched gRNA (fold change relative to a scrambled gRNA, n = 2 biological replicates). (B) Relation between the fold induction of each TF and the basal expression of that TF relative to *GAPDH* expression. (C) Comparison of gRNA enrichment from the single factor CAS-TF screen for two *NEUROG2* gRNAs. (D) Validation of these two *NEUROG2* gRNAs for TF induction and expression of downstream neuronal markers (*p < 0.05 by global one-way ANOVA with a Tukey post hoc test comparing the two *NEUROG2* gRNAs, n = 3 biological replicates, error bars represent SEM). Related to Figures 1 and 2.

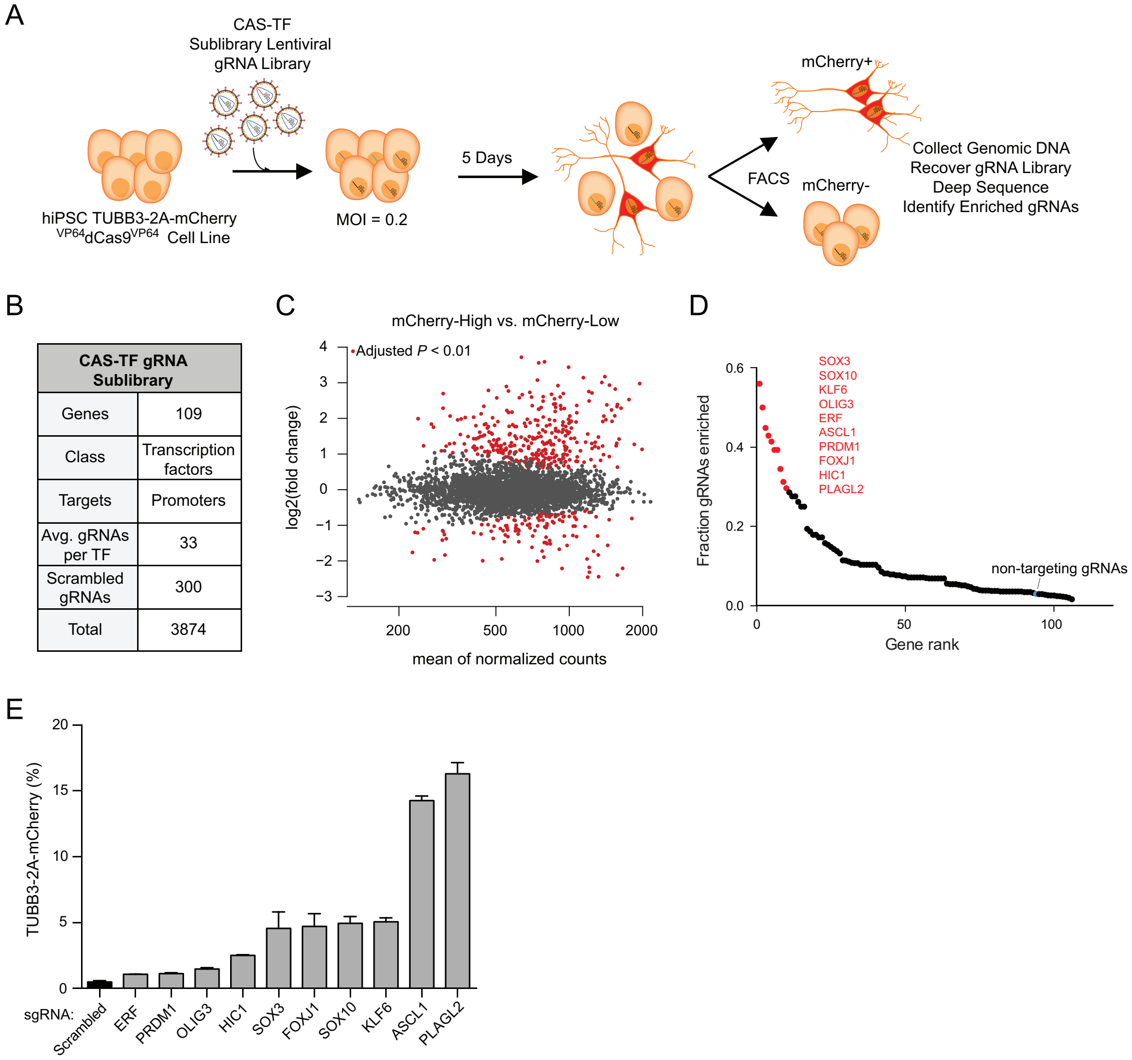

Figure S4. CAS-TF sub-library gRNA screen. (A) Schematic representation of the CRISPRa sub-library screen for neuronal-fate determining transcription factors in human pluripotent stem cells. A ^{VP64}dCas9^{VP64} *TUBB3-2A-mCherry* reporter cell line was transduced with the CAS-TF pooled lentiviral library at an MOI of 0.2 and sorted for mCherry expression via FACS. gRNA abundance in each cell bin was measured by deep sequencing, and depleted or enriched gRNAs were identified by differential expression analysis. (B) The CAS-TF gRNA sub-library was extracted from several previous genome-wide CRISPRa library (Gilbert et al., 2014; Horlbeck, 2016; Konermann et al., 2015; Sanson et al., 2018) and consists of 3,874 gRNAs targeting 109 putative transcription factors (~33 gRNAs per gene). (C) Differential expression analysis of normalized gRNA counts between the mCherry-High and mCherry-Low cell populations. Red data points indicate FDR < 0.01 by differential DESeq2 analysis (n = 3 biological replicates). (D) Ranked list of percent enriched gRNAs per gene. (E) Validations of 10 factors for TUBB3-2A-mCherry expression four days after transduction of gRNAs (n = 2 biological replicates). Related to Figures 1 and 2.

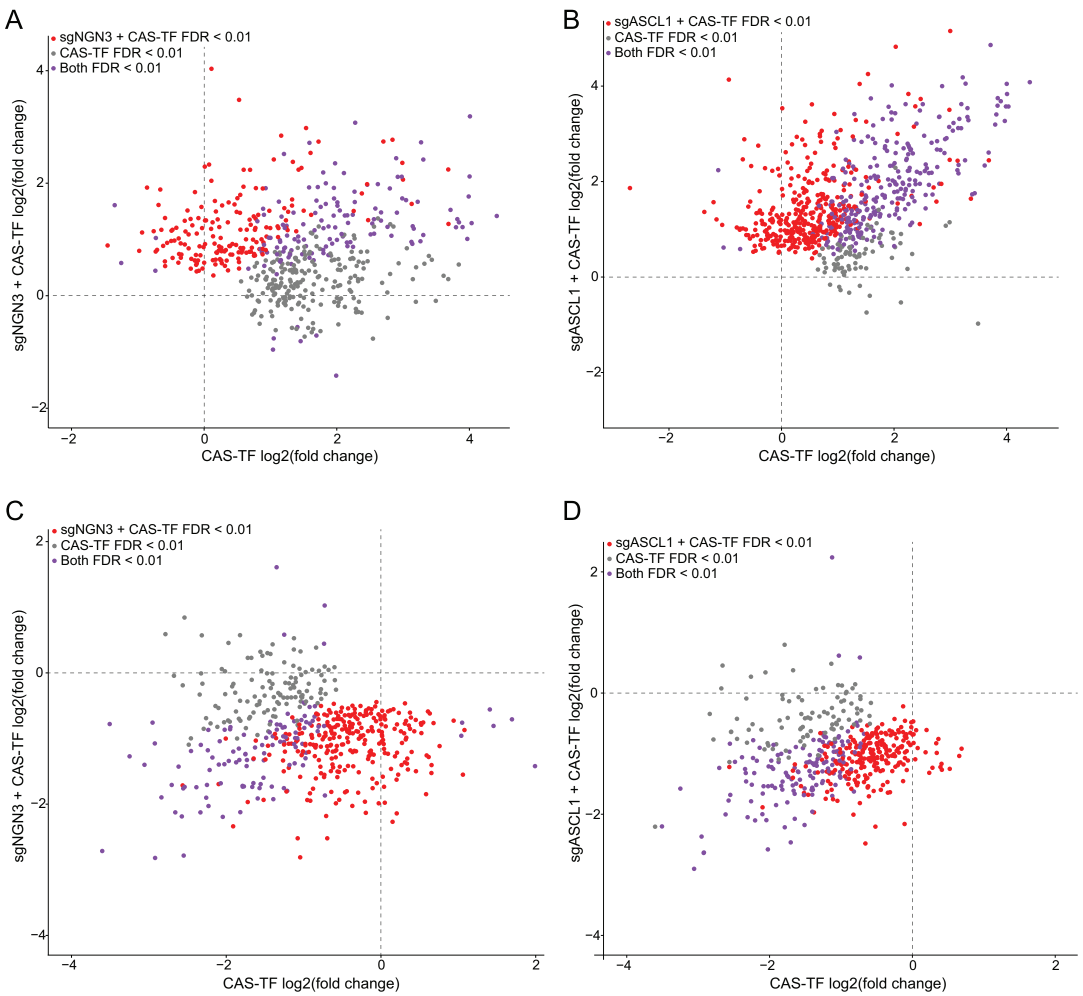

Figure S5. Comparisons of the single factor and paired CAS-TF screens. The fold change in gRNA abundance between mCherry-High and mCherry-Low expressing cells for the (A and B) sgNGN3 versus single factor CAS-TF screens for all positively (A) and negatively (B) enriched gRNAs across both screens and (C and D) sgASCL1 versus single factor CAS-TF screens for all positively (C) and negatively (D) enriched gRNAs across both screens. Related to Figure 3.

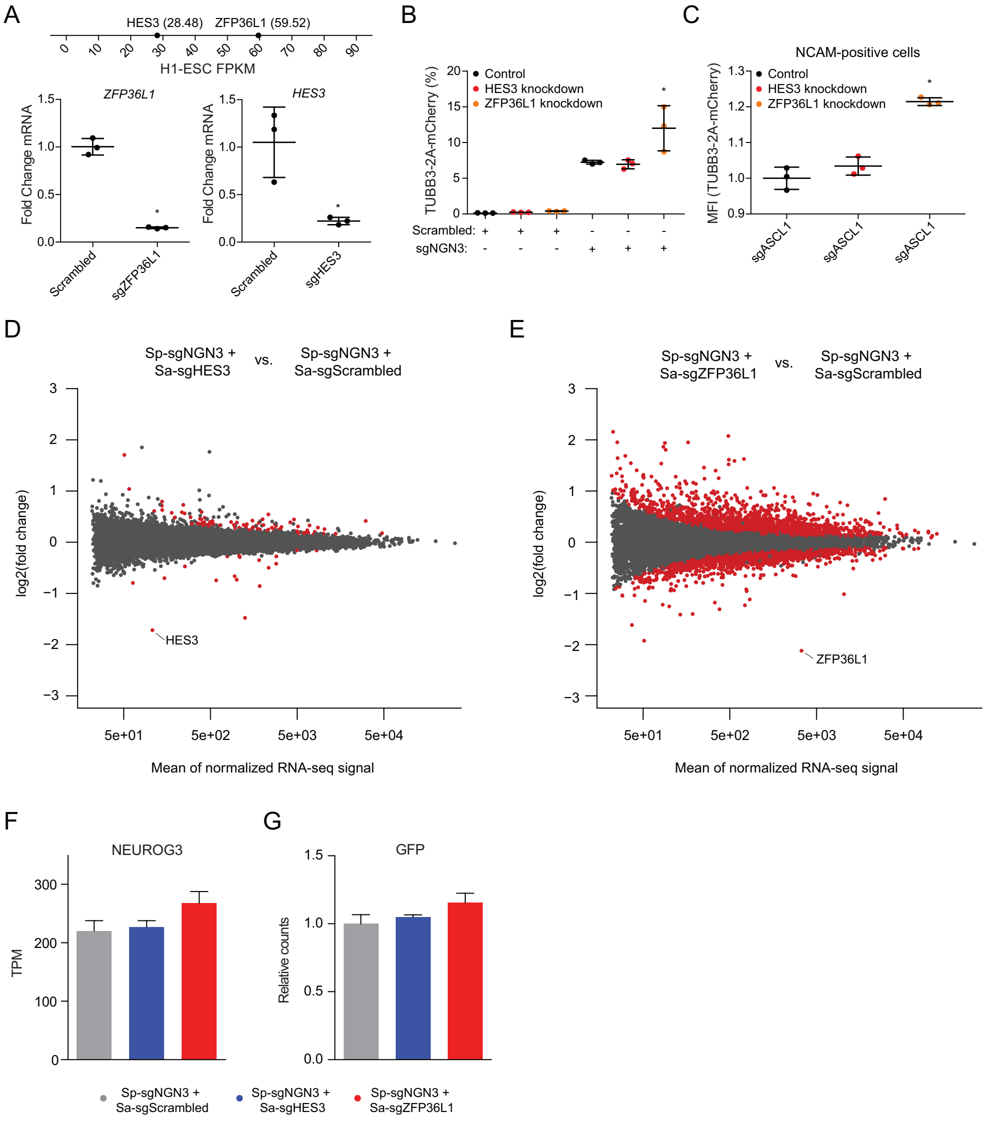

Figure S6. Gene activation and repression with orthogonal CRISPR systems. (A) Targeted repression of *ZFP36L1* and *HES3* in pluripotent stem cells using dSaCas9^{KRAB} targeting the promoter with a single gRNA for seven days (*p < 0.05 by two-tailed t-test, n = 3 biological replicates, error bars represent SEM). Effects on differentiation with either sgNGN3 (B) or sgASLC1 (C) in *ZFP36L1* and *HES3* knockdown cell lines (*p < 0.05 by global one-way ANOVA with Dunnett's post hoc test comparing all groups with either sgNGN3 or sgASCL1 to the Control cell line that received a scrambled non-targeting *S. aureus* gRNA, n = 3 biological replicates, error bars represent SEM). Differential expression analysis for sgNGN3-derived neurons with (D) *HES3* knockdown and (E) *ZFP36L1* knockdown. Red data points indicate FDR < 0.01 by differential expression analysis with DESeq2 (n = 3 biological replicates). (F) Expression of the *S. pyogenes* gRNA target gene, *NEUROG3,* across the three conditions shown. (G) GFP expression on the *S. pyogenes* gRNA lentiviral vector was used as a proxy for transduction level and gRNA expression across the three conditions shown. Related to Figure 6.

Table S1. All positive hits across the three neuronal differentiation screens. Related to Figures 1, 3 and 6.

Single Factor CAS-TF	sgNGN3 + CAS-TF	sgASCL1 + CAS-TF
NEUROG3	PRDM1	RUNX3
SOX4	LHX6	PRDM1
SOX9	NEUROG3	KLF6
KLF4	PAX8	PAX2
NR5A1	SOX3	RFX3
NEUROD1	KLF4	SOX10
SOX17	FLI1	GATA1
SMAD1	FOXH1	KLF5
ATOH1	FEV	KLF1
INSM1	SOX17	ERF
NEUROG1	FOS	LHX6
SOX18	INSM1	PHOX2B
RFX4	SOX2	NANOG
KLF7	WT1	NR5A2
SP8	SOX18	ETV3
OVOL1	ZNF670	NEUROG3
NEUROG2	LHX8	SOX4
ERF (from sublibrary)	OVOL1	SOX9
PRDM1 (from sublibrary)	E2F7	PAX8
OLIG3 (from sublibrary)	AFF1	IRF5
HIC1 (from sublibrary)	HMX2	CDX4
SOX3 (from sublibrary)	MAZ	RARA
FOXJ1 (from sublibrary)	RARA	BHLHE40
SOX10 (from sublibrary)	PROP1	SOX3
KLF6 (from sublibrary)	FOSL1	KLF4
ASCL1 (from sublibrary)	PAX5	NR5A1
PLAGL2 (from sublibrary)	KLF3	IRF4
		ASCL1
		GATA6
		SPIB
		THRB
		FOXH1
		NEUROD1
		SOX17
		CDX2
		ZEB2
		RARG
		INSM1
		FOSL1
		NEUROG1
		SOX1
		WT1

		PAX5
		SOX18
		POU5F1
		RFX4
		KLF7
		NKX2-2
		OVOL2
		FOXJ1
		PRDM14
		VENTX
		LHX8
		GFI1
		KLF17
		OVOL1
		OLIG3
		HMX3
		ZNF521
		ONECUT3
		OVOL3
		ZNF362
		AFF1
		HMX2
		ZNF786
		GATA5
		TBX3
		ZNF385A
		ATOH1
		PROP1
		SOX11
		JUN
		FOXE3
		FERD3L
		E2F7

Table S2. All negative hits across the three neuronal differentiation screens. Related to Figures 1, 3 and 6.

Single Factor CAS-TF	sgNGN3 + CAS-TF	sgASCL1 + CAS-TF
ZIC2	HES2	ETV1
SPI1	SREBF1	ZIC2
GRHL2	CIC	GSC2
TFAP2C	WHSC1	CIC
KLF8	VDR	GRHL2
MYB	HES1	REST
TCF21	ID2	TFAP2C
KLF12	TCF21	SALL1
TWIST1	SNAI1	NFKB1
SNAI1	RREB1	ELF2
RREB1	GCM2	HES1
GCM2	IRF3	MYB
GRHL1	FOXA1	KLF12
ETS1	GATA5	VSX2
BARHL2	GRHL1	NFE2
GRHL3	SOX5	SNAI1
ELF3	DMRT1	TRERF1
PTF1A	GCM1	RREB1
GSX1	BARHL2	IRF1
PBX2	SOX13	IRF3
NOTO	ZEB1	KLF2
KLF3	PITX2	MYOD1
ZNF311	PTF1A	SOX15
ELMSAN1	ZNF282	BARX1
ZNF296	NPAS2	GRHL1
PLEK	ZNF160	SOX5
KMT2A	HES7	ETS1
HES3	ZBED4	SKIL
	SALL4	BARHL2
	GLIS3	SOX13
	TBX22	ERG
	ZNF331	GRHL3
	EGR4	ZNF281
	ZIC5	ELF3
	ZNF710	HESX1
	ZNF697	KLF15
	ZFP36L2	PITX2
	ELMSAN1	PTF1A
	ZNF296	GSX1

	ZNF318	ZNF160
	ZNF570	ETV5
	ZNF683	MYBL1
	ZFP36L1	NOTO
	HES4	DPF1
	ZNF777	MECOM
	HES5	GLIS3
	ZIM2	KLF3
	ZNF579	TBX22
	BMP2	ESX1
	CRAMP1L	ZNF337
	TOX3	ZFP36L2
	FEZF2	ELMSAN1
	HES3	ZNF618
	ZNF791	ZNF296
		ZNF318
		ZNF570
		ZNF497
		ZFP36L1
		HES5
		BMP2
		CRAMP1L
		ZNF821
		KMT2A
		HES3
		BSX

Table S3. Protospacer sequences for all gRNAs used in this study. Related to STAR Methods.

Gene	sgRNA Sequence
Scrambled 1	TGTCGTGATGCGTAGACGG
Scrambled 2	TCATCAAGGAGCATTCCGT
NEUROG3	CTCGAGAGAGCAAACAGAG
RFX4	ATAGAAGGGGGAAGTCGGA
SOX4	CATGCCAAACCCCTCCCCC
NEUROD1	TGAGGGGAGCGGTTGTCGG
INSM1	CGCCGGGCGGGGCGACCAG
KLF7	AGCGCGAGCGCAAGGGACA
SOX9	CTGGGTGACGAGGCGGGAG
SOX17	CAAGGCTACACCTGCCCCC
NEUROG1	TAGCCCGAGCCGACTCCCG
SP8	GCGCGCGCCGTGAGGTCAT
KLF4	CTCCCTTCCATCGTTGCTA
SMAD1	CCGGGCCGGGAATTTGGAG
OVOL1	CGACAGGTAACAAATAGGT
NR5A1	AATACCCCTATCTATCTGG
ATOH1	GCCTGCCCGCGCCCTCCAT
NEUROG2-1	GCAGCGAGGACGAAGGCGG
NEUROG2-2	GGAAAGGCGGTGAAGAAAG
SOX18	GCCTCAGCGGAATCCCGCC
ASCL1	GAGGAGGAGGGGGAGTTTA
ASCL1 (sublibrary)	AATGGAGAGTTTGCAAGGAG
ATOH7	ACTAACACACCATCTGGAG
ATOH8	CGGGGCGGTTGTGCAGGAG
ATOH1-2	GGCTGAGAAGACACGCGAC
ATOH1-3	CACTCGGAGATCACACACC
ATOH1-4	CACGCGACCGGCGCGAGGA
ATOH1-5	TGCGGAGCCGGCTCTCGGC
NR5A1-2	AGAGAAACACCAACAAAGA
NR5A1-3	GGCCTGCAGAGTCACGTGG
NR5A1-4	TGCCCCCACGTGACTCTGC
NR5A1-5	GGGCCACCGGAGGCCCAAT
LHX6	AGGAGGAGGACTACCAAGA
LHX8	CGGGGAACACCGGGCTAAA
E2F7	GCGCCAAGACTCCGAGGGG
RUNX3	CCTGCCGGAGGCCGCCCAA
FOXH1	CCACCCAAAGGCAACTCAG
SOX2	GGATACAAAGGTTTCTCAG
HMX2	AGGCCCTCGGCGCGCTCTG
NKX2.2	CCCTCTAGAGCAAGATGAG
ELMSAN1	GGCGTCCTTAAACCTCAGG
GCM2	ACAGTCCCAGGAACGGAGG

HES1	GTGGACCGCGCCCCCCCAT
HES7	CCCTCTAGGACCCGGCACG
TOX3	AGAAGAGGGGCCCCGGAGA
DMRT1	GGACCCTGCAGCAAAGCCC
BMP2	CCGCCCGCTCGGGGATCCC
ZFP36L1	CTTCCCTACCCGGCGCTTC
ERF	GAGCGTGTGTGTGAGTGCGC
PRDM1	CGGCTGTGCTAGCAATCTGG
OLIG3	GAGCCCTCCTATCTATCCT
HIC1	GCTGTGCGCCGTGCCCGCCC
SOX3	CGGAGGACCCGTGATTGAC
FOXJ1	GCTCGGCTCATTCCCGCCCG
SOX10	CCCTGAGTGTTGGGGATGA
KLF6	TCCCGTGGCTCCCGGCCCGG
PLAGL2	GCCCCGGCCGCTCTAGCCCG
S. aureus Scrambled	TCATCAAGGAGCATTCCGT
S. aureus ZFP36L1	ATGACAACAAGAACCCCGGA
S. aureus HES3	CCCTTCCCCGGGAGGTGTGG

Table S4. All qRT-PCR primers used in this study. Related to STAR Methods.

Gene	Primer Sequence
NCAM Fwd	AACCCAGTGCACCTAAGCTC
NCAM Rev	GGACTTCAGCATGACGTGGT
MAP2 Fwd	CAGCTTGTCTCTAACCGAGGA
MAP2 Rev	TGTGTCGTGTTCTCAAAGGGT
TUBB3 Fwd	TTTGGACATCTCTTCAGGCC
TUBB3 Rev	TTTCACACTCCTTCCGCAC
ZFP36L1 Fwd	CCGAGTCCCCTCACATGTTT
ZFP36L1 Rev	TTGAGTTGTCCAAGGTCGGG
HES3 Fwd	GAAAGTCTCCCTGGCTCGTC
HES3 Rev	CCAAATAGGGAGCGCCTTCA
NEUROG3 Fwd	TTTTCTCCTTTGGGGCTGGG
NEUROG3 Rev	AGGCGTCATCCTTTCTACCG
RFX4 Fwd	GACGAGCGGCCATTCATCAG
RFX4 Rev	CACTCAGTAATCCAGCCGGG
SOX4 Fwd	AACAGGGCGGCTGGTTAATA
SOX4 Rev	ACACTGGTGGCAGGTTAAGG
NEUROD1 Fwd	GATGACTAAGGCTCGCCTGG
NEUROD1 Rev	AGAATAGCAAGGCACCACCT
INSM1 Fwd	TACGCGTTTGTCTCGTGGTT
INSM1 Rev	CAGAGATTGGTAGGCGAGGC
KLF7 Fwd	TTGCATTAGGAGCGAACAGC
KLF7 Rev	AAAAGGGGACTTCTCCACGG
SOX9 Fwd	TAAAACGGTGCTGCTGGGAA
SOX9 Rev	AGTGTGCTCGGGCACTTATT
SOX17 Fwd	GACATGAAGGTGAAGGGCGA
SOX17 Rev	CGTTGTGCAGGTCTGGATTC
NEUROG1 Fwd	AATATCTCCCGGGCGTCTGA
NEUROG1 Rev	GTTCAAGTTGTGCATGCGGT
SP8 Fwd	CTTCTAGGGGAAGAACCGAGG
SP8 Rev	AAGAGGACGAGGAGCGTTTC
KLF4 Fwd	CACCGGACCTACTTACTCGC
KLF4 Rev	AACCCCAAATTGGCCGAGAT
SMAD1 Fwd	GGAGAAAGGAGAGGCCGAGC
SMAD1 Rev	AAAAGTAACCCAGTCAGCACCG
OVOL1 Fwd	GTCCGGCTCGCACTTTAAGA
OVOL1 Rev	CTGAGAACGAGGTCCCTTGC
NR5A1 Fwd	GTGGTGTGAGGGGGTTTCTG
NR5A1 Rev	TACGAATAGTCCATGCCCGC
ATOH1 Fwd	AGGATGCATGGGCTGAACC
ATOH1 Rev	TTGTAGCAGCTCGGACAAGG
NEUROG2 Fwd	CAGGCCAAAGTCACAGCAAC
NEUROG2 Rev	CGATCCGAGCAGCACTAACA

SOX18 Fwd	GCAAAGGACGAGCGCAAG
SOX18 Rev	CTTGTAGTTGGGGTGGTCGC
SOX11 Fwd	AGCGGAGGAGGTTTTCAGTG
SOX11 Rev	TTCCATTCGGTCTCGCCAAA
