# Supplementary material for: Master Regulators and Cofactors of Human Neuronal Cell Fate Specification Identified by CRISPR Gene Activation Screens
Source: Cell Rep. Author manuscript; Available in PMC 2020 Dec 11. (PMC7730023; doi:10.1016/j.celrep.2020.108460)
Supplement: 3 [file NIHMS1651252-supplement-3.pdf]

Cell Reports

Article

Master Regulators and Cofactors of Human Neuronal Cell Fate Specification Identified by CRISPR Gene Activation Screens

Graphical Abstract

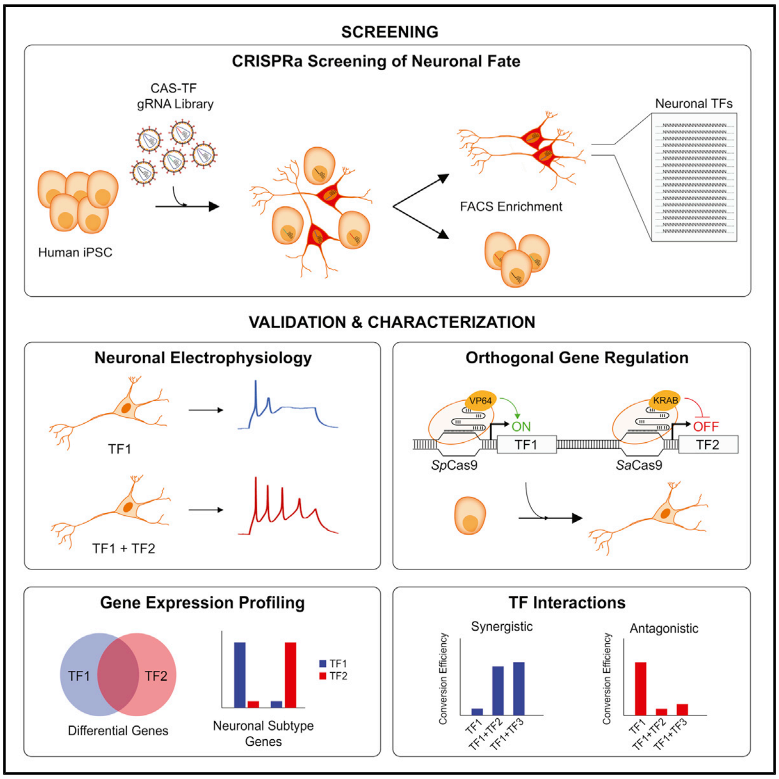

Highlights

- CRISPRa screens identify factors regulating human neuronal fate specification
- Paired gRNA screens reveal synergistic transcription factor interactions
- Identified factors influence conversion rate, subtype profile, and maturation
- Gene regulation with orthogonal CRISPR systems enables improved differentiation

Authors

Joshua B. Black, Sean R. McCutcheon, Shataakshi Dube, ..., Scott H. Soderling, Timothy E. Reddy, Charles A. Gersbach

Correspondence

charles.gersbach@duke.edu

In Brief

Black et al. perform pooled CRISPR activation screens to identify factors that regulate neuronal fate specification of human pluripotent stem cells. The identified factors improve conversion efficiencies and modulate neuronal subtype profiles and maturation. Overall, this approach provides a broad framework for programming complex cellular phenotypes.

Black et al., 2020, Cell Reports *33*, 108460
December 1, 2020 © 2020 The Author(s).
https://doi.org/10.1016/j.celrep.2020.108460

CellPress

Cell Reports

CellPress OPEN ACCESS

Article

Master Regulators and Cofactors of Human Neuronal Cell Fate Specification Identified by CRISPR Gene Activation Screens

Joshua B. Black,[1,2] Sean R. McCutcheon,[1,2] Shataakshi Dube,[3] Alejandro Barrera,[2,4] Tyler S. Klann,[1,2] Grayson A. Rice,[1,2] Shaunak S. Adkar,[2,5] Scott H. Soderling,[3,5] Timothy E. Reddy,[1,2,4,6,7,8] and Charles A. Gersbach[1,2,5,6,7,9,10,*]

[1]Department of Biomedical Engineering, Duke University, Durham, NC 27708, USA
[2]Center for Advanced Genomic Technologies, Duke University, Durham, NC 27708, USA
[3]Department of Neurobiology, Duke University Medical Center, Durham, NC 27710, USA
[4]Department of Biostatistics and Bioinformatics, Duke University Medical Center, Durham, NC 27710, USA
[5]Department of Cell Biology, Duke University Medical Center, Durham, NC 27710, USA
[6]Graduate Program in Computational Biology and Bioinformatics, Duke University, Durham, NC 27708, USA
[7]University Program in Genetics and Genomics, Duke University, Durham, NC 27708, USA
[8]Department of Molecular Genetics and Microbiology, Duke University, Durham, NC 27708, USA
[9]Department of Surgery, Duke University Medical Center, Durham, NC 27710, USA
[10]Lead Contact
*Correspondence: charles.gersbach@duke.edu
https://doi.org/10.1016/j.celrep.2020.108460

SUMMARY

Technologies to reprogram cell-type specification have revolutionized the fields of regenerative medicine and disease modeling. Currently, the selection of fate-determining factors for cell reprogramming applications is typically a laborious and low-throughput process. Therefore, we use high-throughput pooled CRISPR activation (CRISPRa) screens to systematically map human neuronal cell fate regulators. We utilize deactivated Cas9 (dCas9)-based gene activation to target 1,496 putative transcription factors (TFs) in the human genome. Using a reporter of neuronal commitment, we profile the neurogenic activity of these factors in human pluripotent stem cells (PSCs), leading to a curated set of pro-neuronal factors. Activation of pairs of TFs reveals neuronal cofactors, including E2F7, RUNX3, and LHX8, that improve conversion efficiency, subtype specificity, and maturation of neuronal cell types. Finally, using multiplexed gene regulation with orthogonal CRISPR systems, we demonstrate improved neuronal differentiation with concurrent activation and repression of target genes, underscoring the power of CRISPR-based gene regulation for programming complex cellular phenotypes.

INTRODUCTION

Transcription factors (TFs) are fundamental for transmitting complex patterns of intrinsic and extrinsic signals into dynamic gene expression programs that define cell-type identity. Because of their ubiquitous and versatile role across development, homeostasis, and disease, TFs are a common focus for biotechnological applications. For instance, the ectopic overexpression of TFs is sufficient to directly reprogram one cell type into another (Takahashi and Yamanaka, 2016; Vierbuchen and Wernig, 2011; Xu et al., 2015), defining a paradigm to generate clinically relevant cell types for applications in disease modeling, drug discovery, and regenerative medicine.

Recent efforts have been made to catalog the set of all putative human TFs and to define their tissue-specific expression (Lambert et al., 2018; Vaquerizas et al., 2009). While such a catalog provides a useful resource, relatively few TFs have been empirically validated for a role in cell fate specification. Furthermore, the selection of fate-determining TFs for cell reprogramming applications often relies on approaches that evaluate a small subset of TFs (Takahashi and Yamanaka, 2006; Vierbuchen et al., 2010) or use computational models to predict optimal TF combinations (D'Alessio et al., 2015; Morris et al., 2014; Rackham et al., 2016). There remains a need for continued development of high-throughput approaches to systematically profile the causal role of TFs in directing cell-type identity.

CRISPR activation (CRISPRa) screens offer a high-throughput approach to profile thousands of gain-of-function perturbations in a pooled format (Gilbert et al., 2014; Konermann et al., 2015). Genome-wide CRISPRa guide RNA (gRNA) libraries have been designed for improved gRNA activity (Horlbeck et al., 2016; Sanson et al., 2018), and deactivated Cas9 (dCas9)-based activator platforms have been successfully used for cell fate reprogramming in several cell types (Black et al., 2016; Chakraborty et al., 2014; Chavez et al., 2015; Kwon et al., 2020; Liu et al., 2018a, 2018b). Additionally, the capacity for multiplexing and

This is an open access article under the CC BY license (http://creativecommons.org/licenses/by/4.0/).

the orthogonal nature of CRISPR systems enables the study of complex genetic interaction networks that govern cell phenotype (Du et al., 2017; Najm et al., 2018). Unlike open reading frame (ORF) libraries that have been used to profile TF contributions to cell-type identity (Theodorou et al., 2009), CRISPR-based gRNA libraries are more easily designed and scaled and are more amenable to testing combinatorial gene interactions and interrogating the non-coding genome (Klann et al., 2018; Montalbano et al., 2017; Shen et al., 2017). For example, a recent study successfully demonstrated the application of CRISPRa screening to uncover genes involved in cell fate determination of mouse embryonic stem cells (ESCs) (Liu et al., 2018b).

Recent advancements in the throughput of single-cell genomic technologies have facilitated the mapping of neuronal-cell-type diversity in the human brain (Darmanis et al., 2015; Lake et al., 2018). In addition to defining an atlas of neuronal subtypes, these studies have revealed subtype-specific contributions to human disease (Lake et al., 2018; Skene et al., 2018). The generation of these neuronal subtypes *in vitro* at high efficiency and fidelity is essential to elucidate the mechanisms governing neurological diseases and develop novel therapeutic strategies (Mertens et al., 2016).

Here, we developed a CRISPRa screening approach to profile the contribution of all putative human TFs to neuronal cell fate specification of pluripotent stem cells (PSCs). We first performed a single-factor screen to identify master regulators of neuronal fate and identified many known and previously uncharacterized TFs. We subsequently performed paired gRNA screens and identified synergistic and antagonistic TF interactions that enhance or diminish neuronal differentiation, respectively. Importantly, through this method, we have uncovered TFs that increase conversion efficiency and modulate neuronal gene expression programs influencing subtype specificity and maturation of *in-vitro*-derived neurons. More generally, our study provides a framework for identifying the causal role of cell fate regulators in defining any cell type of interest.

RESULTS

Generation of a Human PSC Line for CRISPRa Screening of Neuronal Cell Fate

To enable the enrichment of neuronal cells within a CRISPRa screening framework, we inserted a *2A-mCherry* sequence into exon 4 of the pan-neuronal marker *TUBB3* in a human PSC line (Figure S1A). *TUBB3* is expressed almost exclusively in neurons and is induced early upon the *in vitro* differentiation and reprogramming of cells to neurons (Busskamp et al., 2014; Pang et al., 2011; Vierbuchen et al., 2010). The 2A-mediated ribosomal skipping ensures that mCherry serves as a translational reporter of TUBB3 while also mitigating any interference with endogenous TUBB3 function that might arise from a direct protein fusion.

To enable efficient and robust targeted gene activation in our *TUBB3-P2A-mCherry* cell line, we used a lentiviral vector to establish a clonal cell line expressing dCas9 fused to a VP64 transactivation domain at both its N and C termini (^{VP64}dCas9^{VP64}) under the control of the human ubiquitin C promoter (Kabadi et al., 2014). ^{VP64}dCas9^{VP64} has been used previously to achieve robust endogenous gene activation sufficient for cell fate reprogramming (Black et al., 2016; Chakraborty et al., 2014; Kwon et al., 2020).

To evaluate a CRISPRa approach for neuronal differentiation in our ^{VP64}dCas9^{VP64} *TUBB3-2A-mCherry* cell line, we delivered a pool of four lentiviral gRNAs targeting the proximal promoter of *NEUROG2*, a master regulator of neurogenesis sufficient to generate neurons from PSCs when overexpressed ectopically or when activated endogenously with CRISPRa (Chavez et al., 2015; Zhang et al., 2013). After 5 days of gRNA expression, we detected upregulation of the target gene *NEUROG2*, as well as of the early pan-neuronal markers *NCAM* and *MAP2* (Figure S1B). Targeted gene activation was only achieved if both ^{VP64}dCas9^{VP64} and *NEUROG2* gRNAs were co-expressed (Figure S1B).

Following delivery of *NEUROG2* gRNAs, we detected 15% mCherry-positive cells relative to untreated control cells 6 days after transduction (Figure S1C). To assess the capacity of our *TUBB3-2A-mCherry* reporter cell line to serve as a proxy for a neuronal phenotype, we used fluorescence-activated cell sorting (FACS) to isolate the highest and lowest 10% of mCherry-expressing cells. The mCherry-high cells had higher mRNA expression levels of the mCherry-tagged gene *TUBB3*, as well as *MAP2* (Figure S1D). The *TUBB3-2A-mCherry* cells and CRISPRa approach were used in all screens described in this study.

CRISPRa Screen for Master Regulators of Neuronal Cell Fate

To identify a set of neuronal cell fate regulators in an unbiased manner, we performed a CRISPRa pooled gRNA screen in the *TUBB3-2A-mCherry* cell line (Figure 1A). The gRNA library consisted of gRNAs targeting a set of putative human TFs (Vaquerizas et al., 2009). TFs are essential for cell fate specification and have been applied extensively for cell reprogramming and directed differentiation applications (Xu et al., 2015). We selected a set of 1,496 TFs and constructed a targeted gRNA library of five gRNAs for each transcription start site, extracted from a genome-wide library of optimized CRISPRa gRNAs (Horlbeck et al., 2016) (Figure 1B).

The CRISPRa-TF gRNA lentiviral library (named CRISPRa screen TF [CAS-TF]) was transduced at a multiplicity of infection (MOI) of 0.2 and at 550-fold coverage of the library to ensure that most cells activated a single TF and to account for the stochastic and often inefficient nature of *in vitro* cell differentiations (Figure 1A). After 5 days of gRNA expression, we used FACS to isolate the top and bottom 5% of mCherry-expressing cells (Figure 1C) and quantified gRNA abundance with differential expression analysis following deep sequencing of the protospacers within each sorted bin. Cells were sorted on day 5 post-transduction to permit sufficient time for TF expression and induction of the reporter gene while limiting the loss of post-mitotic neurons with extended time in culture or through passaging. Published examples of induced neurons from TF overexpression often detect TUBB3 expression within 5 days (Black et al., 2016; Busskamp et al., 2014; Vierbuchen et al., 2010).

Compared to a bulk unsorted population of cells, there were gRNAs significantly enriched in the mCherry-high expressing cell bin (false discovery rate [FDR] < 0.01; Figure 1D). We observed

CellPress
OPEN ACCESS

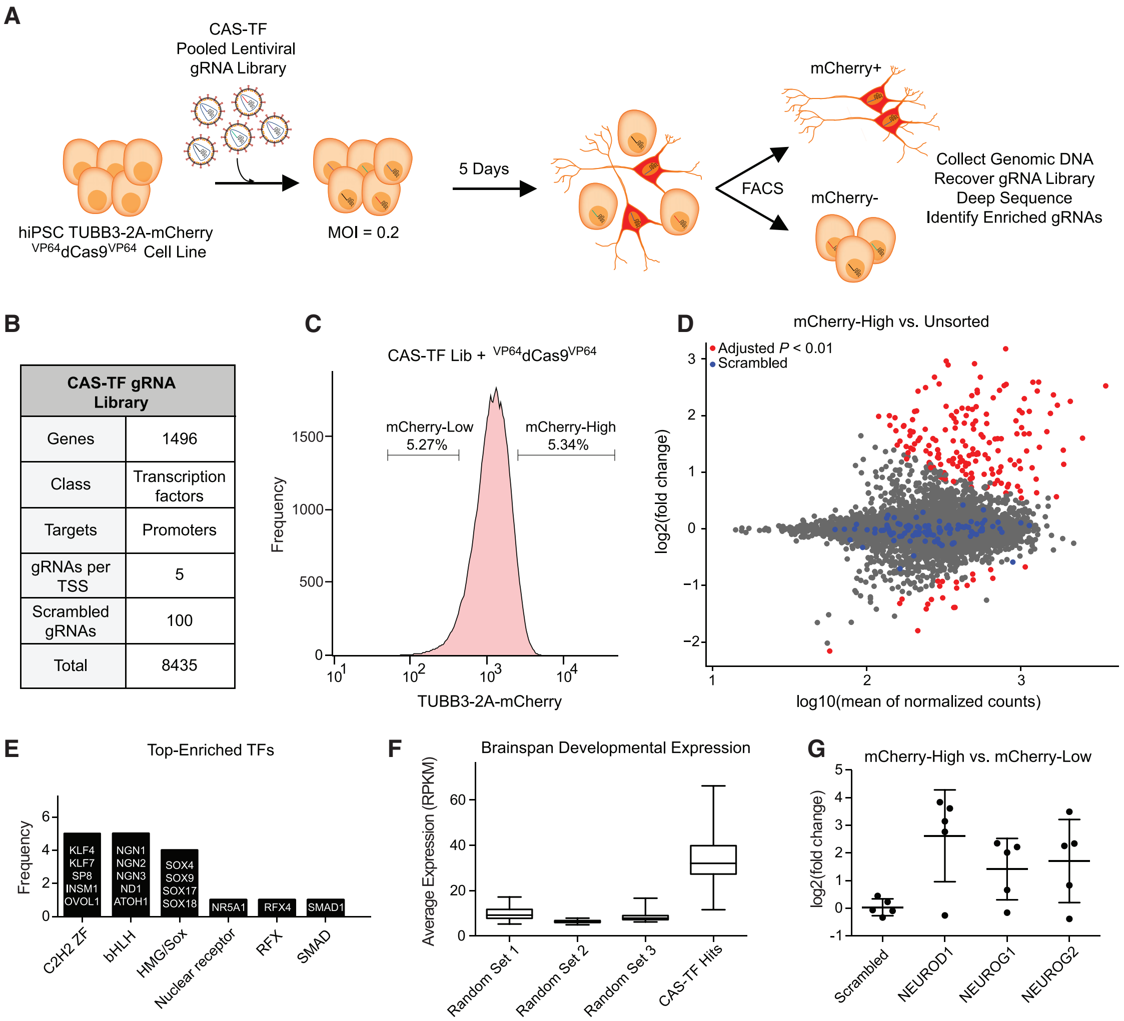

Figure 1. A High-Throughput CRISPRa Screen Identifies Candidate Neurogenic TFs

(A) Schematic representation of a CRISPRa screen for neuronal-fate-determining transcription factors (TFs) in human pluripotent stem cells (PSCs). A ^{VP64}dCas9^{VP64} *TUBB3-2A-mCherry* reporter cell line was transduced with the CAS-TF pooled lentiviral library at an MOI of 0.2 and sorted for mCherry expression via FACS. gRNA abundance in each cell bin was measured by deep sequencing, and depleted or enriched gRNAs were identified by differential expression analysis.

(B) The CAS-TF gRNA library was extracted from a previous genome-wide CRISPRa library (Horlbeck et al., 2016) and consists of 8,435 gRNAs targeting 1,496 putative TFs.

(C) *TUBB3-2A-mCherry* cells were sorted for the highest and lowest 5% of expressing cells based on mCherry signal. A bulk unsorted population of cells was also sampled to establish the baseline gRNA distribution.

(D) Differential expression analysis of normalized gRNA counts between the mCherry-high and unsorted cell populations. Red data points indicate FDR < 0.01 by differential DESeq2 analysis (n = 3 biological replicates). Blue data points indicate a set of 100 scrambled non-targeting gRNAs.

(E) Analysis of TF family type across the 17 TFs identified in the CAS-TF screen.

(F) Comparison of average gene expression (Miller et al., 2014) across multiple developmental time points and anatomical brain regions for the 17 TFs identified in the CAS-TF screen and three randomly generated sets of 17 TFs.

(G) The fold change in gRNA abundance from differential expression analysis between mCherry-High and mCherry-Low cell populations for all five gRNAs from three known proneural TFs compared to a random selection of five scrambled gRNAs.

See also Figure S1.

similar results when comparing mCherry-high- to mCherry-low-expressing cells (Figure S2A). A set of 100 scrambled non-targeting gRNAs were unchanged between the different cell bins (Figure 1D).

The degree of transcriptional activation achieved with dCas9-based activators can vary across a set of gRNAs for a given target gene (Gilbert et al., 2014). As a consequence, we expected to observe a mixture of active and inactive gRNAs for most target genes. Additionally, off-target gRNA activity could promote false positives by modulating reporter gene expression independent of the predicted TF target. To ensure we did not overinterpret the results of a single gRNA, TFs were selected as high-confidence hits if they had at least two gRNAs

significantly enriched in the mCherry-high-expressing cell bin relative to both the unsorted and the mCherry-low cell bins (FDR < 0.01). This approach yielded a list of 17 TFs as candidate neurogenic factors (Figure 1E). The majority of these TFs belonged to C2H2 ZF, basic-helix-loop-helix (bHLH), or high-mobility group (HMG)/Sox DNA-binding domain families, three of the most abundant families across all human TFs (Lambert et al., 2018) (Figure 1E).

We analyzed the expression of the 17 candidate neurogenic factors with publicly available gene expression data in the developing human brain curated as part of BrainSpan (Miller et al., 2014) (http://brainspan.org) We observed that the mean expression of the 17 factors, calculated across several anatomical regions and developmental time points of the human brain (see STAR Methods), was higher than that of a randomly generated set of 17 TFs (Figure 1F).

As a further demonstration of the fidelity of the CAS-TF screen, we observed that three well-characterized proneural factors, *NEUROD1*, *NEUROG1*, and *NEUROG2*, each had several gRNAs enriched in mCherry-high-expressing cells, while a random set of five scrambled non-targeting gRNAs was unchanged (Figure 1G). A fourth gene with expected proneural activity, *ASCL1*, was not selected as a high-confidence hit based on our stringent selection criteria. However, a single *ASCL1* gRNA was enriched in the mCherry-high-expressing cells (Figure S2A), and this gRNA was sufficient to generate mCherry-positive cells expressing *NCAM* and *MAP2* (Figures S2B and S2C).

Validations of Candidate Neurogenic TFs

To validate the activity of the candidate neurogenic TFs, we individually tested the most enriched gRNA for the 17 TFs identified in the CAS-TF screen. We transduced these gRNAs at high MOI into the *TUBB3-2A-mCherry* cell line and evaluated reporter expression after 4 days (Figure 2A). All of the gRNAs tested increased the number of mCherry-positive cells to varying degrees (from ~2% to ~50%) relative to the delivery of a scrambled non-targeting gRNA, although only a subset of 10 factors did so with statistical significance (Figure 2A; $\alpha = 0.05$). To verify CRISPRa activity, we confirmed that all of the TFs were upregulated in response to expression of the appropriate gRNA (Figure S3A). The degree of TF induction directly correlated with the basal expression level of the target gene, consistent with previous reports (Konermann et al., 2015) (Figure S3B).

Further validations of all five gRNAs represented in the CAS-TF library for *ATOH1* and *NR5A1* revealed a direct correlation between the calculated enrichment from the pooled screen and the degree of differentiation assessed with reporter gene expression when the gRNAs were tested individually (Figure 2B). In some cases, gRNAs that were not significantly enriched in the screen were still capable of modest gene activation and neuronal induction (Figures S3C and S3D). For instance, a *NEUROG2* gRNA was sufficient to upregulate *NEUROG2*, which was paralleled by *NCAM* and *MAP2* induction but was not enriched in the CAS-TF screen (Figures S3C and S3D).

Given that we relied on a single reporter gene as a proxy for a neuronal phenotype, we expected that the TFs enriched in the CAS-TF screen would include both master regulators of neuronal fate sufficient to initiate differentiation, as well as cofactors or downstream effectors that only regulate one or a subset of neuronal genes. To clarify these differences within our set of candidate factors, we first evaluated the expression of two other neuronal markers, *NCAM* and *MAP2*, 4 days after gRNA delivery. Several TFs upregulated one or both of these markers, while other TFs generated no change or even downregulation (Figure 2C). For instance, *SOX4*, which induced one of the largest increases in percent mCherry expression at an average of 34%, had no detectable effect on *NCAM* and *MAP2* expression (Figures 2A and 2C).

We used immunofluorescence staining to evaluate the presence of neuronal morphologies with expression of a subset of the TFs identified in our CAS-TF screen (Figure 2D). To ensure robust TF expression and control for differential gRNA activity, we overexpressed cDNAs encoding each TF. Several of the factors, including *NEUROG3* and *NEUROD1*, generated cells with complex dendritic arborization that stained positively for TUBB3 within 4 days of expression (Figure 2D). In contrast, many TFs upregulated TUBB3 as expected but failed to generate cells with neuronal morphologies. We reasoned that the lack of morphological development in these cells could be attributable to slower differentiation kinetics. Other neuronal reprogramming paradigms often require extended culture to achieve morphological maturation (Chanda et al., 2014). To account for this, we further cultured the cells for 11 days with primary astrocytes and found that with extended culture time, *ATOH1*, *ATOH7*, and *ASCL1* were sufficient to generate cells with complex neuronal morphologies that stained positively for MAP2 (Figure 2E). We did not observe similar morphological maturation with prolonged culture for *KLF7*, *NR5A1*, and *OVOL1*.

To account for variation in response to expression of these TFs across different PSC lines and see if the lack of complete neuronal differentiation for several factors was a cell-line-specific phenomenon, we also tested *KLF7*, *NR5A1*, and *OVOL1* in H9 ESCs. We similarly observed a clear upregulation of TUBB3 without the development of neuronal morphologies (Figure 2F). As expected, *NEUROG3* was able to induce rapid differentiation with the development of clear neuronal morphologies.

While the 17 high-confidence TF hits had a high validation rate, we suspected that many proneural TFs, similar to *ASCL1*, did not meet our stringent cutoff criteria. In fact, there were 109 other TFs that contained at least a single gRNA significantly enriched in the mCherry-high-expressing cells but were not called as a hit. To further investigate these TFs, we first focused on TFs who shared a subfamily with one of the 17 high-confidence hits. For instance, *ATOH1* was a high-confidence hit with several enriched gRNAs; however, *ATOH7* and *ATOH8* both had only a single enriched gRNA (Figure S2A). When these gRNAs were tested individually, *ATOH7* and *ATOH8* were both sufficient to generate mCherry-positive cells expressing *NCAM* and/or *MAP2* (Figures S2B and S2C), indicating that many hits with only single enriched gRNAs by this cutoff represent true positives.

In order to more comprehensively validate the activity of these 109 TFs, we performed a secondary sub-library screen targeting only these TFs (Figure S4). This screen was performed in an

CellPress
OPEN ACCESS

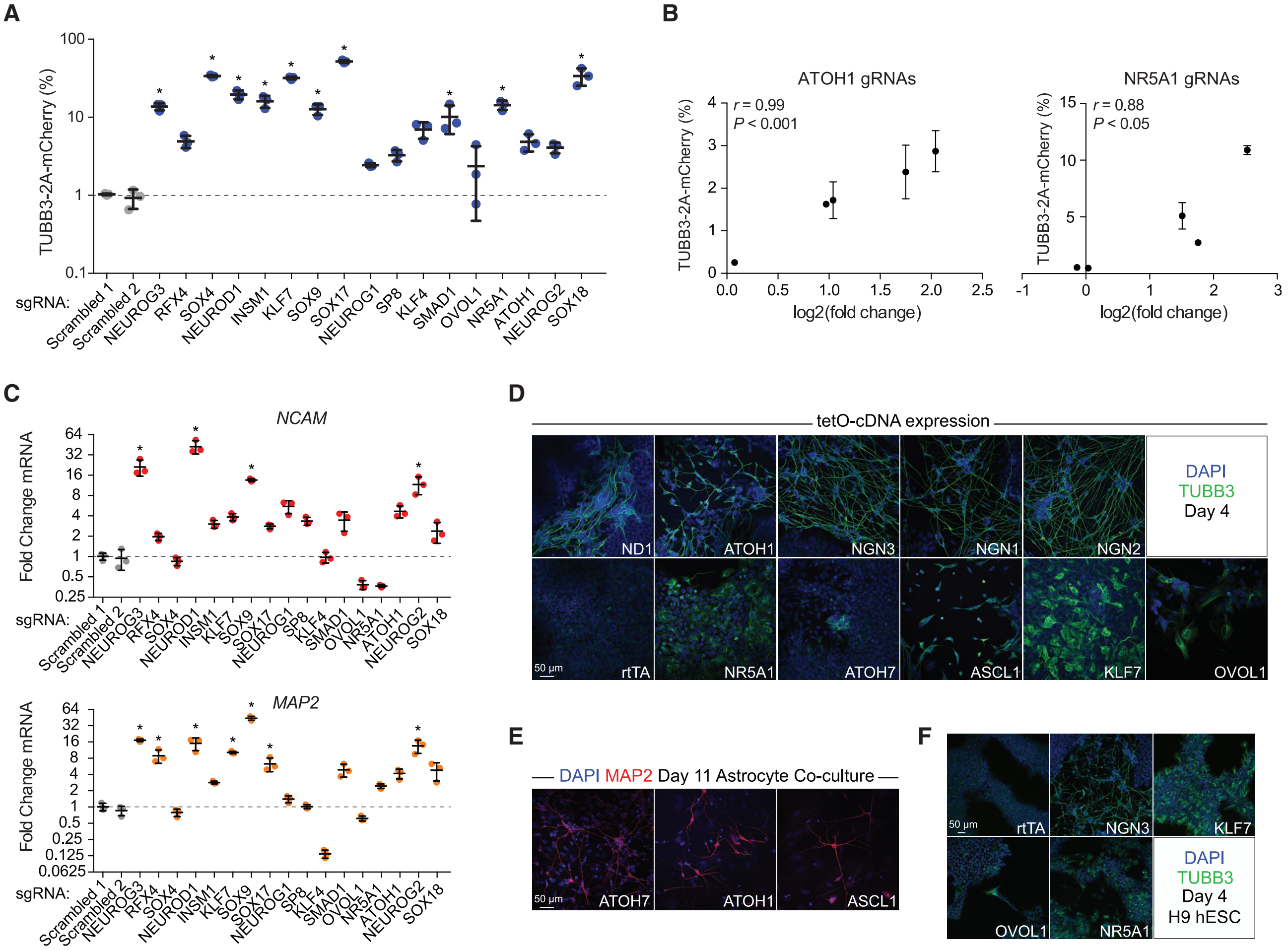

Figure 2. Many Candidate Factors Generate Neuronal Cells from PSCs

(A) Validations of 17 factors for TUBB3-2A-mCherry expression 4 days after transduction of gRNAs (*$p < 0.05$ by global one-way ANOVA with Dunnett's post hoc test comparing all groups to Scrambled 1, gating set to 1% positive for scrambled gRNAs; n = 3 biological replicates; error bars represent SEM).

(B) The relationship between TUBB3-2A-mCherry expression assessed by individual validations and the fold change in gRNA abundance from differential expression analysis of the library selection for all five gRNAs from *ATOH1* and *NR5A1*.

(C) Validations of 17 factors for the induction of the pan-neuronal markers *NCAM* (top) and *MAP2* (bottom) 4 days after transduction of gRNAs (*$p < 0.05$ by global one-way ANOVA with Dunnett's post hoc test comparing all groups to Scrambled 1; n = 3 biological replicates; error bars represent SEM).

(D) Immunofluorescence staining of iPSCs assessing TUBB3 expression 4 days after transduction with tetracycline-inducible lentiviral vectors carrying cDNAs encoding the indicated factors, or with a M2rtTA-only negative control. Scale bar, 50 μm.

(E) Immunofluorescence staining of iPSCs assessing MAP2 expression with the indicated factors after extended co-culture with astrocytes. Scale bar, 50 μm.

(F) Immunofluorescence staining of H9 human ESCs (hESCs) assessing TUBB3 expression 4 days after transduction of the indicated factors. Scale bar, 50 μm.

See also Figures S2–S4.

identical fashion to the first CAS-TF screen (Figure S4A), but the new sub-library consisted of an average of 33 gRNAs per TF (Figure S4B). This screen revealed additional gRNAs enriched in mCherry-high cells (Figure S4C). However, the majority of genes in the sub-library had relatively few enriched gRNAs, similar to a pool of scrambled non-targeting gRNAs (Figure S4D). A few genes had over 40% of gRNAs enriched in the mCherry-high bin. However, individual validations of these gRNAs revealed mostly subtle effects on the mCherry reporter (Figure S4E). This analysis both informs the design of robust CRISPRa screens and confirms that our screen design was successful in identifying the most robust neurogenic factors.

Paired gRNA Screens Identify Neuronal Cofactors

TFs often function cooperatively to orchestrate gene expression programs (Chronis et al., 2017). Similarly, TF-mediated cell reprogramming often benefits from the co-expression of combinations of TFs to improve conversion efficiencies, maturation, and subtype specification (Wapinski et al., 2013; Yang et al., 2017). Because the mechanisms underlying the improvements observed with co-expressed TFs are often unknown, and because effective cofactors can have minimal activity when expressed alone, it can be challenging to predict effective TF cocktails. To address this challenge, we performed pooled screens

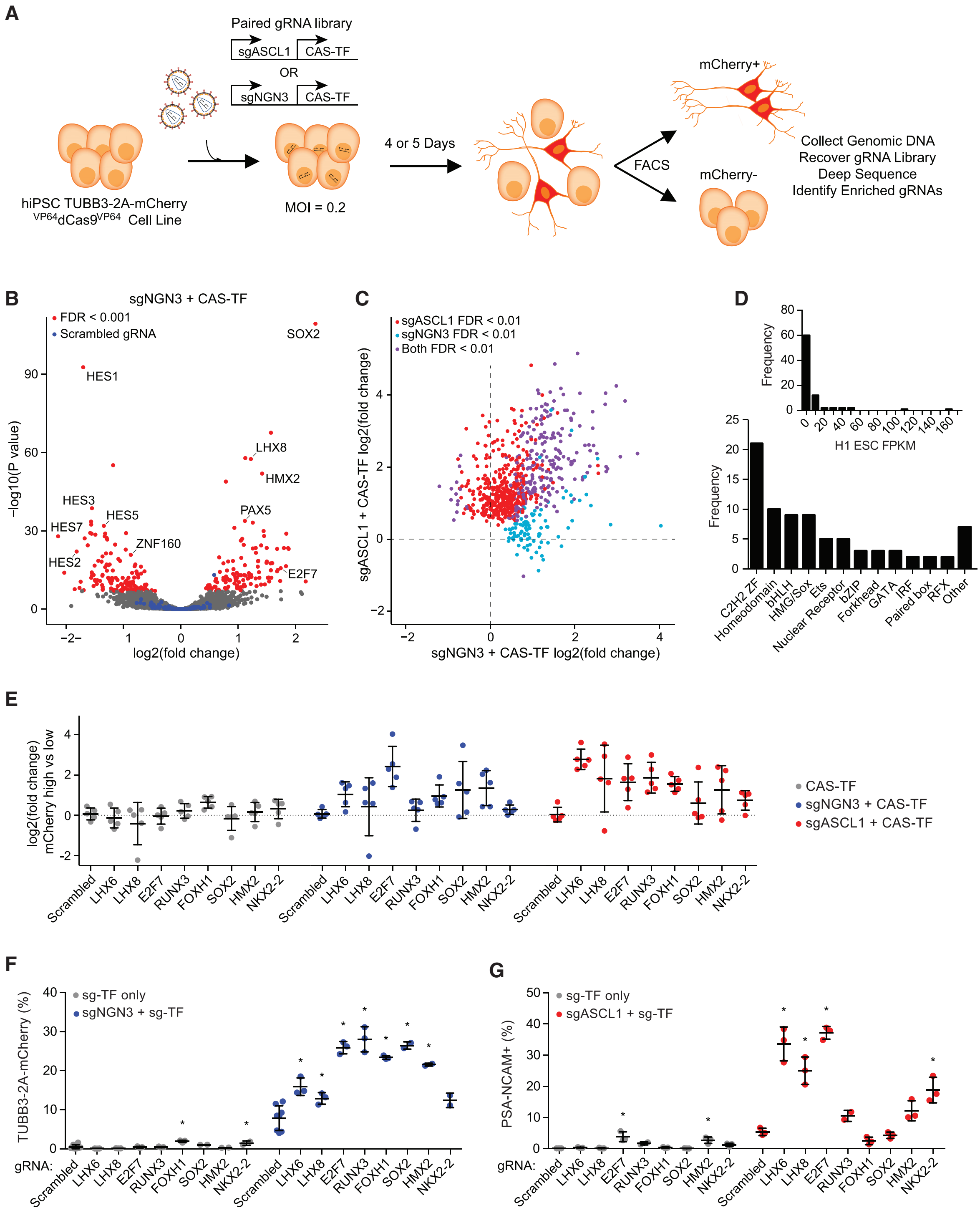

Figure 3. Paired gRNA Screens Identify Cofactors of Neuronal Differentiation

(A) Schematic representation of paired CRISPRa screens for neuronal-fate-determining TFs in human PSCs. A dual gRNA expression vector was used to co-express a neurogenic factor with the CAS-TF gRNA library. Two independent screens were performed with sgASCL1 and sgNGN3.

(B) A volcano plot of significance (p value) versus fold change in gRNA abundance based on differential DESeq2 analysis between mCherry-high and unsorted cell populations for the sgNGN3 paired screen. Red data points indicate FDR < 0.001 (n = 3 biological replicates). Blue data points indicate a set of 100 scrambled non-targeting gRNAs.

(C) The fold change in gRNA abundance for the sgASCL1 versus sgNGN3 paired screens for all positively enriched gRNAs across both screens.

(D) Analysis of TF family type and basal expression level in PSCs (Consortium, 2012) for the positive hits from both paired screens.

(legend continued on next page)

with pairs of gRNAs to identify combinations of regulators that modulate neuronal differentiation of human PSCs.

We hypothesized that some co-regulators of neuronal differentiation would lack detectable activity when expressed on their own and thus would not be identified in our initial single-factor CAS-TF screen. Rather, these cofactors might require pairing with another neurogenic factor to reveal their activity. To enable the identification of such TFs, we opted to perform screens pairing a validated neurogenic TF identified from the single-factor screen with the remaining CAS-TF library (Figure 3A). Two such independent screens were performed with a single gRNA for either *NEUROG3* (sgNGN3) or *ASCL1* (sgASCL1) (Figure 3A). A pair of gRNAs was co-expressed on a single lentiviral vector from two independent RNA polymerase III promoters in a format adapted from a previous study (Adamson et al., 2016). *NEUROG3* and *ASCL1* were chosen due to their strong neurogenic activity but differing kinetics of differentiation (Figures 2D and 2E). The paired screens were performed as described for the single-factor screen, with each cell now receiving a single pair of gRNAs.

Due to the constitutive presence of a validated neurogenic factor in each cell, a clear mCherry-positive cell population emerged. Because of this basal neurogenic stimulus, in addition to the detection of positive cofactors of differentiation, we were also able to readily detect negative regulators in the mCherry-low-expressing cells (Figure 3B).

Effective cofactors that enhance conversion efficiency are often shared across different neuronal reprogramming paradigms but can contribute to subtype specification in context-dependent ways (Tsunemoto et al., 2018). Similarly, we hypothesized that many cofactors would be shared between *NEUROG3* and *ASCL1*. Consistent with this hypothesis, we found that the majority of positive regulators were shared between the two screens (Figure 3C). However, there were several factors enriched uniquely when combined with either *NEUROG3* or *ASCL1* (Figure 3C). For example, *FEV* was positively enriched with *NEUROG3* only, whereas *NKX2.2* was positively enriched with *ASCL1* only. Importantly, both the sgNGN3 and sgASCL1 screens identified TFs that were not observed in the single-factor CAS-TF screen (Figure S5). Many of these TFs, including *LHX6*, *LHX8*, and *HMX2*, are implicated in neuronal development and subtype specification (Flandin et al., 2011; Wylie et al., 2010) but have not been extensively characterized for the *in vitro* generation of neurons. A list of all candidate neurogenic factors identified across all three screens can be found in Table S1.

The positive hits from the two paired CAS-TF screens encompassed a diverse set of TF families (Figure 3D). The majority of these TFs were not expressed or lowly expressed in PSCs; however, several factors were more highly expressed (Consortium, 2012) (Figure 3D). A set of eight TFs were chosen for further validations. These TFs were predicted to have minimal activity on their own but enhanced neurogenic activity when co-expressed with *NEUROG3* and/or *ASCL1* (Figure 3E). While this subset of eight TFs was selected for further characterization, there are numerous other candidate factors revealed by the CRISPRa paired screens that could be subject to future studies (Table S1).

All of the TFs tested improved the conversion efficiency to mCherry-positive cells up to 3-fold when paired with sgNGN3 compared to sgNGN3 co-expressed with a scrambled gRNA (Figure 3F). Because sgASCL1 only increased the mCherry reporter to modest levels on its own, we chose to use NCAM staining for the gRNA validations for the pairings with this gRNA. Only *E2F7* and *HMX2* had modest effects on NCAM expression on their own (Figure 3G). However, several of the TFs significantly increased the neurogenic activity of *ASCL1*, including up to 8-fold for *E2F7* (Figure 3G). Consistent with the predicted outcomes from the screens, *NKX2.2* had a significant effect with *ASCL1*, but not with *NEUROG3* (Figures 3E–3G).

Neurogenic TFs Modulate Subtype Specificity and Maturation

Neuronal subtype identity and degree of synaptic maturation are important features defining the utility of *in-vitro*-derived neurons for disease modeling and cell therapy applications. Consequently, the development of protocols to improve maturation kinetics and purity of neuronal subtypes has been a primary focus in the field. Given the diversity of neurogenic TFs identified through our CRISPRa screens and the range of conversion efficiencies observed through validation experiments, we reasoned that many of these TFs likely influence subtype identity and maturation in distinct ways. To begin to address this question, we performed bulk mRNA sequencing to more globally assess the degree of neuronal conversion and compare the transcriptional diversity in neuronal populations generated with different TFs.

We started by analyzing neurons derived from a single TF. While combinations of TFs often enhance the specificity of subtype generation and improve the conversion efficiency and maturation kinetics, single TFs can be sufficient to generate functional neurons with subtype proclivity (Chanda et al., 2014; Teratani-Ota et al., 2016; Zhang et al., 2013). We chose to first perform mRNA sequencing on neurons derived from either *ATOH1* or *NEUROG3* overexpression (Figure 4). These TFs had some of the highest conversion efficiencies determined through validation experiments (Figure 2), which facilitates the isolation of sufficient material for sequencing. Additionally, while the neurogenic activity of both *ATOH1* and *NEUROG3* has been confirmed previously (Sagal et al., 2014; Tsunemoto et al., 2018; Xue et al., 2019), our understanding of the role of *ATOH1* and *NEUROG3* in *in vitro* neuronal differentiation remains incomplete.

We overexpressed the cDNAs encoding either *ATOH1* or *NEUROG3*, used FACS to purify TUBB3-mCherry-positive cells, and performed mRNA sequencing after 7 days of transgene

(E) The fold change in gRNA abundance for a set of TFs predicted to have no activity individually and synergistic activity in the sgASCL1 and sgNGN3 paired screens.

(F and G) Validations of TF cofactors for sgNGN3 with TUBB3-2A-mCherry (F) and sgASCL1 with NCAM staining (G).

*$p < 0.05$ by global one-way ANOVA with Dunnett's post hoc test comparing all groups to scrambled 1; n = 3 biological replicates; error bars represent SEM. See also Figure S5.

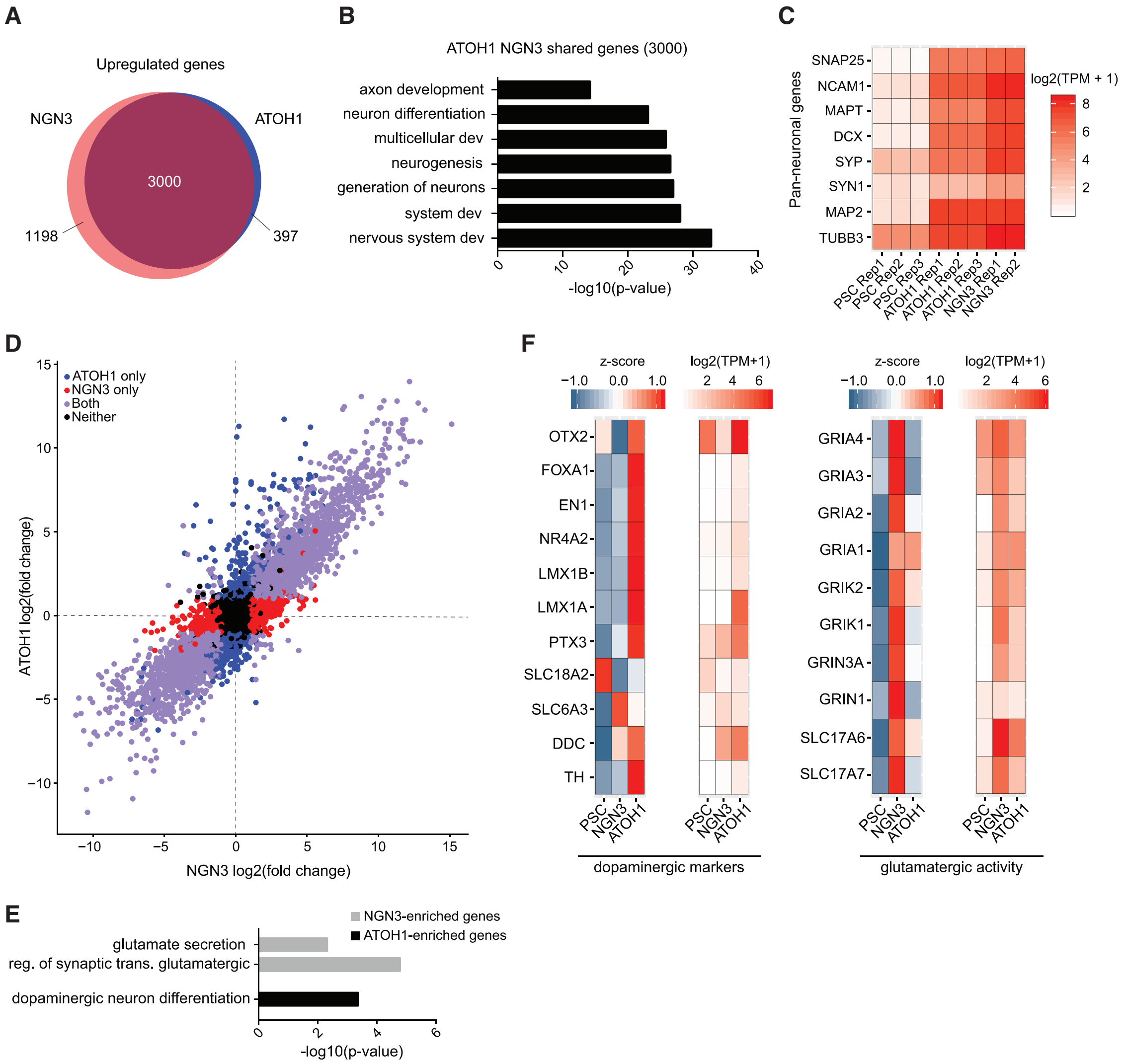

Figure 4. Transcriptional Diversity of Neurons Generated by Single TFs

(A) Differentially upregulated genes detected in *ATOH1* and *NEUROG3*-derived neurons (FDR < 0.01 and log2(fold change) >1 relative to control iPSCs).

(B) Enriched Gene Ontology (GO) terms for the set of 3,000 genes shared and upregulated between *ATOH1* and *NEUROG3*.

(C) Expression level (log2(TPM + 1)) of a set of pan-neuronal genes across all replicate samples analyzed.

(D) Comparison of all detected genes between *ATOH1* and *NEUROG3*-derived neurons. Red and blue circles represent genes differentially expressed with either *NEUROG3* or *ATOH1*, respectively.

(E) GO term analysis for markers upregulated uniquely with either *NEUROG3* or *ATOH1*.

(F) Expression level (log2(TPM + 1)) and corresponding *Z* scores for a set of dopaminergic and glutamatergic markers.

expression. Both populations of neurons had over 3,000 genes upregulated relative to the starting population of undifferentiated PSCs (Figure 4A). The set of shared genes was enriched in Gene Ontology (GO) terms associated with neuronal differentiation and development (Figure 4B). Importantly, a set of pan-neuronal genes was highly enriched across all replicates for *ATOH1* (three replicates) and *NEUROG3* (two replicates) relative to PSCs (Figure 4C).

Surprisingly, we observed a strong correlation across all detectable genes between *ATOH1*- and *NEUROG3*-derived neurons, indicating a striking consistency in the induction of the core neuronal program and suppression of the pluripotency network (Figure 4D). However, a subset of genes was more highly expressed with either *ATOH1* or *NEUROG3* (Figure 4D). These genes were enriched in GO terms related to glutamatergic activity for *NEUROG3* and dopaminergic activity for *ATOH1* (Figure 4E). Indeed, when we examined a set of markers expected of the two neuronal subtypes, we found clear enrichment in dopaminergic markers for *ATOH1* and glutamatergic markers for *NEUROG3* (Figure 4F). While certain canonical markers of dopaminergic neurons, such as tyrosine hydroxylase (TH), remained lowly expressed, many TFs associated with dopaminergic specification, such as *LMX1A*, were more highly expressed in *ATOH1*-derived neurons (Figure 4F).

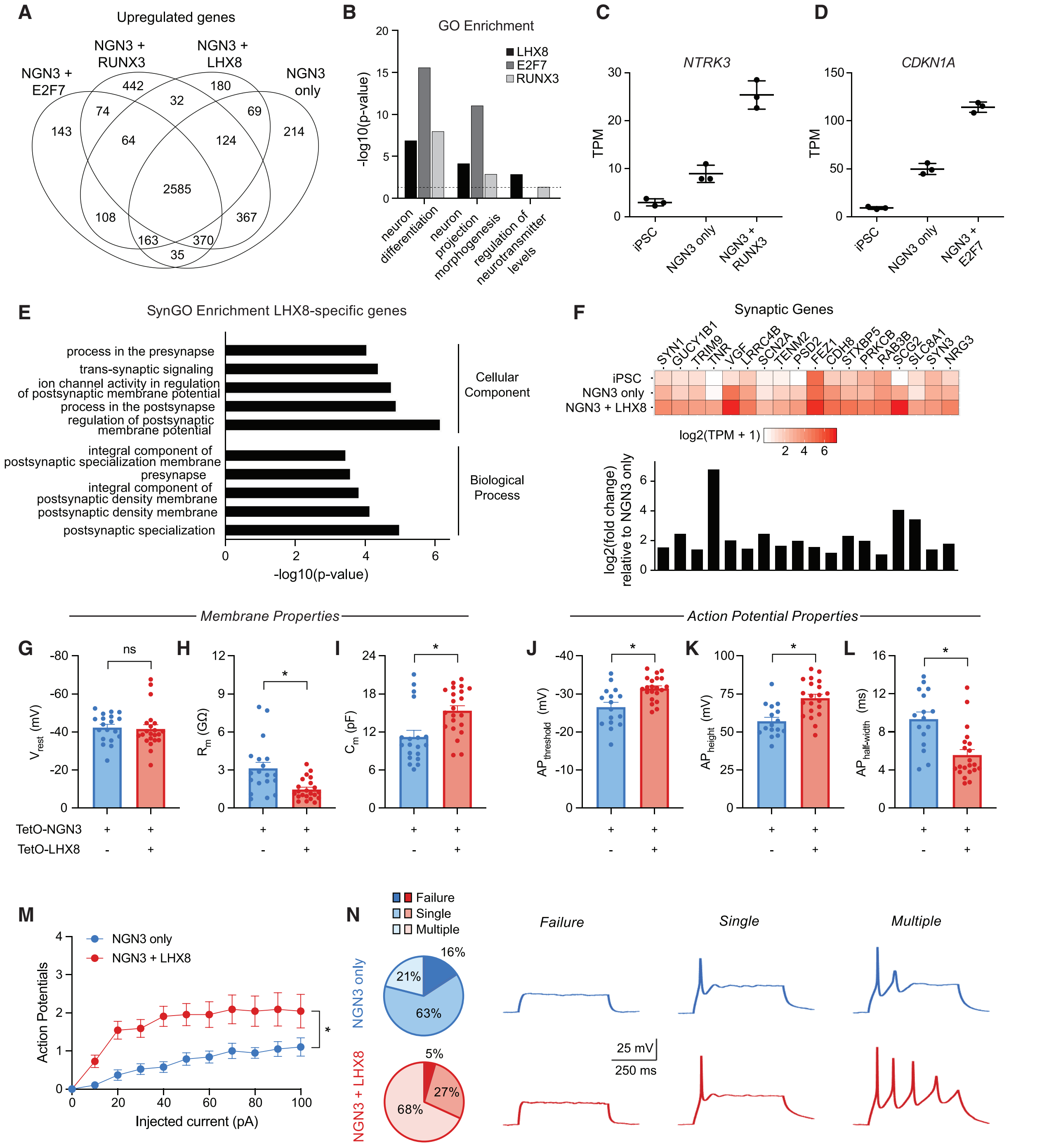

Figure 5. Transcriptional and Functional Maturation of Neurons Generated with Pairs of TFs

(A) Differentially upregulated genes detected in neurons derived from pairs of TFs (FDR < 0.01 and log2(fold change) > 1 relative to control iPSCs).

(B) GO terms enriched in the set of differentially upregulated genes with pairs of TFs compared to *NEUROG3* alone.

(C and D) Upregulation of (C) *NTRK3* and (D) *CDKN1A* with the addition of *RUNX3* or *E2F7,* respectively.

(E) SynGO terms for the set of genes differentially upregulated with the addition of *LHX8*.

(F) Expression level (bottom: log2(fold change); top: log2(TPM + 1)) for a set of synaptic markers.

(G–I) Average values of membrane properties, including resting membrane potential (V_{rest}) (G), input resistance (R_m) (H), and membrane capacitance (C_m) (I) for day 7 neurons generated with *NEUROG3* alone or in combination with *LHX8*.

(J–L) Average values of action potential properties, including action potential threshold ($AP_{threshold}$) (J), action potential height (AP_{height}) (K), and action potential half-width ($AP_{half\text{-}width}$) (L) for day 7 neurons generated with *NEUROG3* alone or in combination with *LHX8*.

(legend continued on next page)

In many cases, combinations of TFs can aid in the precision of neuronal subtype specification or enhance conversion efficiency and maturation. We reasoned that the cofactors identified in our paired gRNA screens would serve as prime candidates for modulating subtype identity and maturation when combined with neurogenic factors identified in the single-factor screen. Consequently, we chose to perform mRNA sequencing on neurons derived from *NEUROG3* alone or in combination with *E2F7*, *RUNX3*, or *LHX8*. These three cofactors were preferentially selected due to their substantial influence on differentiation efficiency assessed through gRNA validations (Figure 3). We chose *NEUROG3* due to its defined preference for generating glutamatergic neurons, often considered a default subtype. We overexpressed the cDNAs encoding *NEUROG3* alone or in combination with *E2F7*, *RUNX3*, or *LHX8* and performed mRNA sequencing after 6 days of transgene expression.

Similar to the *ATOH1* and *NEUROG3* comparison, all TF pairs shared a core set of upregulated genes (Figure 5A). However, genes uniquely upregulated with each TF pair relative to *NEUROG3* alone were enriched in GO terms related to neuronal differentiation and development, consistent with the previously measured increase in TUBB3 expression and improvements in conversion efficiency with expression of these neuronal cofactors (Figure 5B).

Importantly, each TF pair uniquely upregulated genes related to specification and maturation of particular neuronal subtypes. For example, the addition of *RUNX3* led to an increase in expression of *NTRK3*, encoding the TrkC neutrophin-3 receptor linked to the development of proprioceptive dorsal root ganglion neurons (Figure 5C) (Ernsberger, 2009). The addition of *E2F7* led to an increase in *CDKN1A,* encoding the p21 cell-cycle regulator involved in neuronal fate commitment and morphogenesis (Figure 5D) (Kreis et al., 2019). A subset of genes more highly expressed with the addition of *LHX8* were enriched in synaptic GO (SynGO) (Koopmans et al., 2019) terms associated with synaptic development, a hallmark of neuronal maturation (Figure 5E). In agreement with the GO term analysis, a set of genes related to synapse development, regulation, and function were clearly upregulated with the addition of *LHX8* (Figure 5F).

To evaluate if the addition of *LHX8* influenced the electrophysiological maturation of *NEUROG3*-derived neurons, we performed patch-clamp recordings of TUBB3-2A-mCherry-positive cells 7 days after transgene induction. While we did not observe a difference in the resting membrane potential (Figure 5G), we did observe a decrease in membrane resistance (Figure 5H) and an increase in membrane capacitance (Figure 5I) with the addition of *LHX8* relative to *NEUROG3* alone. Several metrics of action potential maturation were improved with *LHX8*, including a decrease in firing threshold (Figure 5J), an increase in action potential height (Figure 5K), and a decrease in action potential half-width (Figure 5L). Additionally, neurons with *LHX8* fired action potentials at higher frequency for a given step depolarization with current injection (Figure 5M) and had a higher proportion of recorded cells that fired multiple actions potentials (Figure 5N). Cells generated with *NEUROG3* alone more frequently failed to fire or only fired a single low-amplitude action potential (Figure 5N).

Paired gRNA Screens Identify Negative Regulators of Neuronal Fate

The conversion efficiencies achieved with cell reprogramming and differentiation protocols often vary depending on the starting and ending cell types (Vierbuchen and Wernig, 2012). Generally, more distantly related cell types, or more aged cell lines, are less amenable to conversion (Ahlenius et al., 2016). For instance, the reprogramming of astrocytes to neurons is often more efficient than that of fibroblasts to neurons, with efficiencies further reduced in adult fibroblasts relative to embryonic fibroblasts (Gascón et al., 2017). These discrepancies in reprogramming outcomes can in part be explained by variation in gene expression profiles and epigenetic states of cells of different type or developmental age (Wapinski et al., 2013). Consequently, this cellular context can create a barrier preventing proper TF activity, reducing conversion efficiency and fidelity.

High-throughput loss-of-function RNAi screens have been instrumental in the identification of molecular barriers preventing cell-type reprogramming and influencing conversion efficiencies (Cheloufi et al., 2015). Importantly, ablation of such barriers often results in significant improvements in reprogramming outcomes (Cheloufi et al., 2015). Through our paired CRISPRa screens, we identified TFs whose activation impeded neuronal differentiation (Figures 3B). These candidate negative regulators included several members of the HES gene family of canonical neuronal repressors downstream of Notch signaling, in addition to many other uncharacterized TFs. A list of all candidate negative regulators identified across all three screens can be found in Table S2.

Interestingly, the majority of the negative regulators were shared across the sgNGN3 and sgASCL1 screens (Figure 6A). They consisted of a diverse set of TFs across many TF families with a wide range of basal expression in ESCs (Consortium, 2012). When tested individually with single gRNAs co-expressed with a *NEUROG3* gRNA, several of the TFs, including *HES1* and *DMRT1*, reduced the percentage of mCherry-positive cells back to basal levels (Figure 6B). To prove that this repression was not confined to only the reporter gene, we also demonstrated reductions in *NCAM* expression up to 8-fold with seven of the eight repressive factors tested (Figure 6C). We similarly observed repression of neuronal differentiation when these factors were tested in H9 human ESCs (hESCs) (Figure 6D). In fact, there was a striking correlation between the relative influence of these

(M) Average number of action potentials generated with respect to amplitude of injected current (*$p < 0.05$, two-way ANOVA).

(N) Example traces of cells with failed (left), single (middle), or multiple (right) action potentials. The corresponding pie chart represents the total fraction of cells analyzed that failed to generate an action potential (dark shade), generated a single action potential (medium shade), or generated multiple action potentials (light shade) in response to a single depolarization current injection.

For (G)–(L), ns, not significant; *$p < 0.05$, unpaired t test (if data passes normality; alpha = 0.05) or Mann-Whitney test (if data fails normality; alpha = 0.05); n = 19 cells for *NEUROG3* alone; n = 22 cells for *NEUROG3* + *LHX8*.

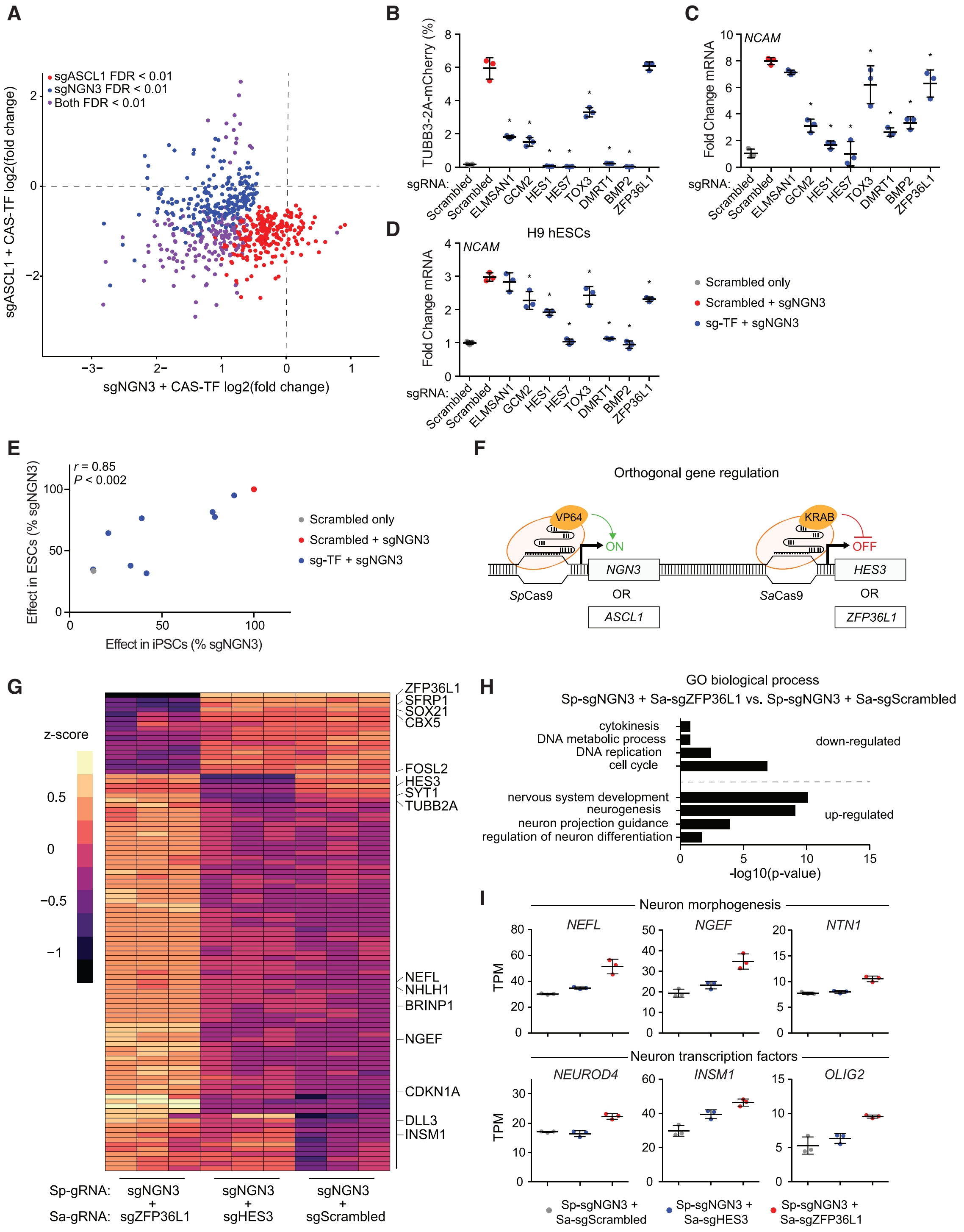

Figure 6. Paired gRNA Screens Identify Negative Regulators of Neuronal Differentiation

(A) The fold change in gRNA abundance for the sgASCL1 versus sgNGN3 paired screens for all negatively enriched gRNAs across both screens.

(B and C) Validations for a subset of TFs measuring percent TUBB3-2A-mCherry-positive cells (B) and expression of the pan-neuronal marker *NCAM* (C) (*$p < 0.05$ by global one-way ANOVA with Dunnett's post hoc test comparing all groups to the sgNGN3 + scrambled gRNA condition; n = 3 biological replicates; error bars represent SEM).

(D) Validations of the negative regulators in H9 hESCs.

(E) Comparison of gRNA effects on neuronal differentiation in iPSCs versus ESCs.

(F) Schematic representation of orthogonal gene activation and repression.

(legend continued on next page)

negative regulators in iPSCs versus ESCs (Figure 6E), underscoring the robustness of these effects across multiple PSC lines.

We reasoned that some of these identified negative regulators that were expressed basally in PSCs may serve as barriers to neuronal conversion, and that their inhibition could improve differentiation efficiency. Cas9 proteins from different bacterial species can be programmed for orthogonal gene regulation and epigenetic modification (Esvelt et al., 2013; Gao et al., 2016). Therefore, we chose to use the orthogonal dSaCas9^{KRAB} (Thakore et al., 2018), based on the Cas9 protein from *S. aureus*, to target the promoters of two negative regulators expressed basally in PSCs, *ZFP36L1* and *HES3* (Figure 6F). Targeting the promoters of these genes with dSaCas9^{KRAB} led to transcriptional repression of 10-fold and 4-fold for *ZFP36L1* and *HES3*, respectively (Figure S6A).

The use of dSaCas9^{KRAB} for targeted gene repression enables the co-expression of the orthogonal ^{VP64}dSpCas9^{VP64} for concurrent activation of a neurogenic factor (Figure 6F). *TUBB3-2A-mCherry* ^{VP64}dSpCas9^{VP64} iPSCs were first transduced with a dSaCas9^{KRAB} lentivirus that co-expresses a *ZFP36L1*, *HES3*, or scrambled *S. aureus* gRNA. 9 days after transduction of *S. aureus* gRNAs, cells were transduced with a lentivirus encoding either sgNGN3 or sgASCL1 from *S. pyogenes* and analyzed 4 days after this final transduction. Knockdown of *ZFP36L1* increased the percent mCherry-positive cells obtained with sgNGN3 2-fold relative to a control cell line expressing a scrambled *S. aureus* gRNA (Figure S6B). Similarly, *ZFP36L1* knockdown increased the mCherry reporter gene expression level 1.2-fold in the NCAM-positive population of differentiating cells obtained with sgASCL1 (Figure S6C).

To identify the genome-wide effects of this orthogonal CRISPR-based regulation, we performed mRNA sequencing on neurons derived from *NGN3* activation concurrent with repression of *ZFP36L1* or *HES3*. While knockdown of *HES3* resulted in only a few subtle changes in gene expression relative to cells that received a scrambled *S. aureus* gRNA (Figure S6D), knockdown of *ZFP36L1* led to a significant change in the global gene expression profile (Figures 6G and S6E) relative to activation of *NGN3* alone. We did also observe a subtle increase in expression of *NEUROG3* and of the *S. pyogenes* gRNA, quantified by expression of a GFP transgene on the gRNA vector, in *ZFP36L1* knockdown cells (Figures S6F and S6G). Genes upregulated in neuronal cells with *ZFP36L1* knockdown were enriched in GO terms related to neuronal differentiation and morphological development (Figure 6H). In contrast, genes downregulated with *ZFP36L1* knockdown were enriched in GO terms related to cell-cycle development and progression (Figure 6H). Examples of genes upregulated with *ZFP36L1* knockdown include the neuronal TFs *NEUROD4*, *INSM1*, and *OLIG2*, as well as genes involved in neuronal morphogenesis, including *NEFL*, *NGEF*, and *NTN1* (Figure 6I).

DISCUSSION

In this study, we systematically profiled 1,496 putative human TFs for their role in regulating neuronal differentiation of PSCs through single and paired CRISPRa screens. This work underscores the utility of CRISPR-based technologies for perturbing gene expression in a high-throughput manner and highlights the robust nature of dCas9-based gene activation for studying the causal role of gene expression in complex cellular phenotypes.

The use of an early pan-neuronal marker like *TUBB3* as a proxy for a neuronal phenotype enabled the identification of a broad set of TFs with varying neurogenic activity. For instance, while *NEUROG3* was sufficient to rapidly generate neuronal cells within 4 days of expression, *ATOH7* and *ASCL1* required more extended time in culture to achieve a similar phenotype (Figures 2D and 2E). It is likely that the addition of cofactors, like those identified in our paired gRNA screens, could improve the efficiency and kinetics of differentiation as seen with other cell reprogramming studies (Pang et al., 2011). Additionally, several TFs, including *KLF7*, *NR5A1*, and *OVOL1*, induced the expression of *TUBB3* but failed to generate neuronal cells (Figure 2D). These TFs might serve as cofactors or downstream regulators that require the co-expression of other neurogenic factors to obtain a more complete differentiation. Indeed, many of the TFs identified in the single-factor screen were also hits in the paired gRNA screens (Table S1).

We found that several TFs with clear neurogenic activity, including *ASCL1* and *ATOH7*, had only a single gRNA enriched in the CAS-TF screen (Figure S2). Because a single enriched gRNA could be the result of off-target activity or noise, it is challenging to accurately classify these gRNAs. The use of more gRNAs per gene or improved dCas9-based activators might help to more accurately define true-positive effects. Indeed, our sub-library screen with a greater number of gRNAs per gene revealed several additional candidate hits (Figure S4). Further improvements in gRNA design (Sanson et al., 2018) and screen analysis (Daley et al., 2018) will continue to make CRISPR-based screens more robust and extensible to more complex phenotypes.

Through the use of paired gRNA screens, we identified a set of TFs that improved neuronal differentiation efficiency, maturation, and subtype specification. Interestingly, the majority of these TFs did not possess neurogenic activity on their own, as assessed in our single-factor CAS-TF screen. This observation underscores the importance of synergistic TF interactions that govern cell differentiation and supports the use of unbiased methods to identify these TFs. In our study, we identified *E2F7* as improving neuronal conversion efficiency (Figures 3F and 3G), possibly due to its known role in inhibiting cell proliferation (Westendorp et al., 2012), an important switch in the conversion from proliferative PSCs to post-mitotic neurons (Gascón et al., 2017). Additionally, we found that *RUNX3* uniquely induced subtype-specific receptor

(G) Relative expression of the top 100 variable genes quantified by *Z* score among all three groups tested.
(H) GO terms enriched in the set of differentially expressed genes in sgNGN3-derived neurons with *ZFP36L1* knockdown.
(I) Example set of differentially expressed genes associated with neuronal differentiation and morphological development.
See also Figure S6.

gene expression (Figure 5C) and thus could be a useful addition to differentiation protocols to more precisely guide neuronal subtype identity. The neuronal cofactor *LHX8* had a profound influence on markers of neuronal maturation, as seen with the enrichment of many synapse-related genes and clear improvements in electrophysiological maturation (Figure 5). Functional synapse formation is an essential phenotype for *in-vitro*-derived neurons, and it is often the rate-limiting step (Chanda et al., 2014). Improving synaptic maturation through TF programming could serve to expedite the development of useful neuronal models for disease modeling and drug screening.

Future studies may take advantage of alternative screening modalities to further characterize cell-lineage-specifying factors. For example, a more comprehensive list of neuronal TFs may have been identified by performing screens that relied on multiple neuronal markers or used markers of maturation or subtype identity. Alternatively, rather than assaying for a few discrete markers, these screens could be performed with a single-cell RNA-sequencing (scRNA-seq) output to more accurately define the diversity of neuronal phenotypes obtained with different TF combinations and benchmark these results against the growing atlas of scRNA-seq data from human neurons (Keil et al., 2018; Parekh et al., 2018; Tian et al., 2019). The TFs identified from the screens in our study serve as prime candidates for sub-libraries to test in these alternative approaches that may be more limited in the scale of library size.

The paired gRNA screens also identified negative regulators of neuronal differentiation. Knockdown of one of those TFs, *ZFP36L1*, was sufficient to improve differentiation, leading to global changes in gene expression toward a more differentiated neuronal phenotype (Figures 6G–6I). While the effects on differentiation were somewhat modest in this example, more dramatic improvements might be seen in cell types that are less amenable to conversion, such as adult aged fibroblasts (Ahlenius et al., 2016). Importantly, many of the negative regulators identified in our screens are expressed in other cell types used for reprogramming studies, such as fibroblasts and astrocytes.

A recent study using CRISPRa screens to identify neuronal regulators in mouse PSCs found that overexpression of the epigenetic modifying enzyme *Ezh2* was sufficient to generate neurons, and it modulated the efficiency of neuronal conversion when paired with other neurogenic factors (Liu et al., 2018b). Surprisingly, there was little overlap between the neurogenic factors identified in our screen and those determined through the similar study in mouse cells (Liu et al., 2018b). While this could be attributable to technical differences in experimental approach, it also likely highlights the inherent differences in the plasticity of mouse versus human cells. Mouse cells are commonly more amenable to reprogramming, often obtaining higher efficiencies of conversion and shortened time to maturation (Pang et al., 2011; Vierbuchen et al., 2010). Consequently, human cells often require additional factors in order to achieve comparable conversion outcomes to their mouse counterparts (Pang et al., 2011).

Additional CRISPRa screens targeting epigenetic modifiers or other gene subsets besides TFs will help to further elucidate the extent to which gene activation can modulate neuronal cell fate. The continued development of synthetic systems for programmable regulation of endogenous gene expression and chromatin state (Erwin et al., 2017; O'Geen et al., 2019), and the application of these systems to more complex *in vitro* and *in vivo* models (Eguchi et al., 2016; Xu and Heller, 2019), will enable studies to more comprehensively define the gene networks and epigenetic mechanisms that govern cell fate decisions.

Overall, through this study, we have identified a broad set of TFs that control neuronal fate specification in human cells. We hope that this catalog of factors will serve as a basis for the development of protocols for the generation of diverse neuronal cell types for applications in regenerative medicine and disease modeling. Ultimately, the CRISPRa screening platform detailed in this study is extensible to other cell reprogramming paradigms and can facilitate the *in vitro* production of other clinically relevant cell types.

STAR★METHODS

Detailed methods are provided in the online version of this paper and include the following:

- KEY RESOURCES TABLE
- RESOURCE AVAILABILITY
  - Lead Contact
  - Materials Availability
  - Data and Code Availability
- EXPERIMENTAL MODEL AND SUBJECT DETAILS
  - Cell Culture
  - Construction of a TUBB3-2A-mCherry pluripotent stem cell line
- METHOD DETAILS
  - Plasmid construction
  - Lentiviral production and titration
  - CAS-TF gRNA library design and cloning
  - Single and paired CAS-TF neuronal differentiation screens
  - Sub-library screen
  - gRNA library sequencing
  - *In vivo* expression comparison
  - gRNA and cDNA validations
  - Quantitative RT-PCR
  - Immunofluorescence staining
  - RNA-sequencing with tetO cDNA expression
  - Electrophysiology
  - Orthogonal CRISPR-based gene regulation
- QUANTIFICATION AND STATISTICAL ANALYSIS
  - Data processing and enrichment analysis for CRISPRa screens
  - RNA-sequencing analysis
  - Statistical methods

SUPPLEMENTAL INFORMATION

Supplemental Information can be found online at https://doi.org/10.1016/j.celrep.2020.108460.

ACKNOWLEDGMENTS

The authors thank the Gersbach lab members for technical assistance and helpful discussions. This work was supported by National Institutes of Health

(NIH) grants (R21NS103007, DP2OD008586, R01DA036865, UM1HG009428, R01HG010741, and U01AI146356), an Allen Distinguished Investigator Award from the Paul G. Allen Frontiers Group, the Open Philanthropy Project, and a National Science Foundation (NSF) grant (EFMA-1830957). J.B.B. was supported by a NIH predoctoral fellowship (F31NS105419) and an NIGMS biotechnology training grant (T32GM008555). S.D. was supported by an NSF graduate research fellowship (DGE-1644868).

AUTHOR CONTRIBUTIONS

J.B.B., S.R.M., S.D., G.A.R., and C.A.G. designed experiments. J.B.B., S.R.M., S.D., G.A.R., and S.S.A. performed the experiments. J.B.B. and T.S.K. analyzed the data from the CRISPRa screens. J.B.B., A.B., and T.E.R. analyzed the RNA-sequencing data. S.D. performed patch-clamp recordings. S.H.S, T.E.R., and C.A.G. supervised the study. J.B.B. and C.A.G. wrote the manuscript with contributions by all authors.

DECLARATION OF INTERESTS

J.B.B., T.S.K., T.E.R., and C.A.G. are named inventors on patent applications related to genome engineering technologies. T.S.K., T.E.R., and C.A.G. are co-founders of Element Genomics. C.A.G. is a co-founder of Tune Therapeutics and Locus Biosciences and is an advisor to Tune Therapeutics, Sarepta Therapeutics, Levo Therapeutics, and Iveric Bio. J.B.B. and T.S.K. are co-founders and employees of Tune Therapeutics.

Received: August 11, 2019
Revised: August 2, 2020
Accepted: November 9, 2020
Published: December 1, 2020

REFERENCES

Adamson, B., Norman, T.M., Jost, M., Cho, M.Y., Nunez, J.K., Chen, Y., Villalta, J.E., Gilbert, L.A., Horlbeck, M.A., Hein, M.Y., et al. (2016). A multiplexed single-cell CRISPR screening platform enables systematic dissection of the unfolded protein response. Cell *167*, 1867–1882.e1821.

Ahlenius, H., Chanda, S., Webb, A.E., Yousif, I., Karmazin, J., Prusiner, S.B., Brunet, A., Südhof, T.C., and Wernig, M. (2016). FoxO3 regulates neuronal reprogramming of cells from postnatal and aging mice. Proc. Natl. Acad. Sci. USA *113*, 8514–8519.

Black, J.B., Adler, A.F., Wang, H.G., D'Ippolito, A.M., Hutchinson, H.A., Reddy, T.E., Pitt, G.S., Leong, K.W., and Gersbach, C.A. (2016). Targeted epigenetic remodeling of endogenous loci by CRISPR/Cas9-based transcriptional activators directly converts fibroblasts to neuronal cells. Cell Stem Cell *19*, 406–414.

Busskamp, V., Lewis, N.E., Guye, P., Ng, A.H., Shipman, S.L., Byrne, S.M., Sanjana, N.E., Murn, J., Li, Y., Li, S., et al. (2014). Rapid neurogenesis through transcriptional activation in human stem cells. Mol. Syst. Biol. *10*, 760.

Chakraborty, S., Ji, H., Kabadi, A.M., Gersbach, C.A., Christoforou, N., and Leong, K.W. (2014). A CRISPR/Cas9-based system for reprogramming cell lineage specification. Stem Cell Reports *3*, 940–947.

Chanda, S., Ang, C.E., Davila, J., Pak, C., Mall, M., Lee, Q.Y., Ahlenius, H., Jung, S.W., Südhof, T.C., and Wernig, M. (2014). Generation of induced neuronal cells by the single reprogramming factor ASCL1. Stem Cell Reports *3*, 282–296.

Chavez, A., Scheiman, J., Vora, S., Pruitt, B.W., Tuttle, M., P R Iyer, E., Lin, S., Kiani, S., Guzman, C.D., Wiegand, D.J., et al. (2015). Highly efficient Cas9-mediated transcriptional programming. Nat. Methods *12*, 326–328.

Cheloufi, S., Elling, U., Hopfgartner, B., Jung, Y.L., Murn, J., Ninova, M., Hubmann, M., Badeaux, A.I., Euong Ang, C., Tenen, D., et al. (2015). The histone chaperone CAF-1 safeguards somatic cell identity. Nature *528*, 218–224.

Chen, B., Gilbert, L.A., Cimini, B.A., Schnitzbauer, J., Zhang, W., Li, G.W., Park, J., Blackburn, E.H., Weissman, J.S., Qi, L.S., and Huang, B. (2013). Dynamic imaging of genomic loci in living human cells by an optimized CRISPR/Cas system. Cell *155*, 1479–1491.

Chronis, C., Fiziev, P., Papp, B., Butz, S., Bonora, G., Sabri, S., Ernst, J., and Plath, K. (2017). Cooperative binding of transcription factors orchestrates reprogramming. Cell *168*, 442–459.e420.

Consortium, E.P.; ENCODE Project Consortium (2012). An integrated encyclopedia of DNA elements in the human genome. Nature *489*, 57–74.

D'Alessio, A.C., Fan, Z.P., Wert, K.J., Baranov, P., Cohen, M.A., Saini, J.S., Cohick, E., Charniga, C., Dadon, D., Hannett, N.M., et al. (2015). A systematic approach to identify candidate transcription factors that control cell identity. Stem Cell Reports *5*, 763–775.

Daley, T.P., Lin, Z., Lin, X., Liu, Y., Wong, W.H., and Qi, L.S. (2018). CRISPhieRmix: a hierarchical mixture model for CRISPR pooled screens. Genome Biol. *19*, 159.

Darmanis, S., Sloan, S.A., Zhang, Y., Enge, M., Caneda, C., Shuer, L.M., Hayden Gephart, M.G., Barres, B.A., and Quake, S.R. (2015). A survey of human brain transcriptome diversity at the single cell level. Proc. Natl. Acad. Sci. USA *112*, 7285–7290.

Dobin, A., Davis, C.A., Schlesinger, F., Drenkow, J., Zaleski, C., Jha, S., Batut, P., Chaisson, M., and Gingeras, T.R. (2013). STAR: ultrafast universal RNA-seq aligner. Bioinformatics *29*, 15–21.

Du, D., Roguev, A., Gordon, D.E., Chen, M., Chen, S.H., Shales, M., Shen, J.P., Ideker, T., Mali, P., Qi, L.S., and Krogan, N.J. (2017). Genetic interaction mapping in mammalian cells using CRISPR interference. Nat. Methods *14*, 577–580.

Eguchi, A., Wleklinski, M.J., Spurgat, M.C., Heiderscheit, E.A., Kropornicka, A.S., Vu, C.K., Bhimsaria, D., Swanson, S.A., Stewart, R., Ramanathan, P., et al. (2016). Reprogramming cell fate with a genome-scale library of artificial transcription factors. Proc. Natl. Acad. Sci. USA *113*, E8257–E8266.

Ernsberger, U. (2009). Role of neurotrophin signalling in the differentiation of neurons from dorsal root ganglia and sympathetic ganglia. Cell Tissue Res. *336*, 349–384.

Erwin, G.S., Grieshop, M.P., Ali, A., Qi, J., Lawlor, M., Kumar, D., Ahmad, I., McNally, A., Teider, N., Worringer, K., et al. (2017). Synthetic transcription elongation factors license transcription across repressive chromatin. Science *358*, 1617–1622.

Esvelt, K.M., Mali, P., Braff, J.L., Moosburner, M., Yaung, S.J., and Church, G.M. (2013). Orthogonal Cas9 proteins for RNA-guided gene regulation and editing. Nat. Methods *10*, 1116–1121.

Flandin, P., Zhao, Y., Vogt, D., Jeong, J., Long, J., Potter, G., Westphal, H., and Rubenstein, J.L. (2011). Lhx6 and Lhx8 coordinately induce neuronal expression of Shh that controls the generation of interneuron progenitors. Neuron *70*, 939–950.

Gao, Y., Xiong, X., Wong, S., Charles, E.J., Lim, W.A., and Qi, L.S. (2016). Complex transcriptional modulation with orthogonal and inducible dCas9 regulators. Nat. Methods *13*, 1043–1049.

Gascón, S., Masserdotti, G., Russo, G.L., and Götz, M. (2017). Direct neuronal reprogramming: achievements, hurdles, and new roads to success. Cell Stem Cell *21*, 18–34.

Gilbert, L.A., Horlbeck, M.A., Adamson, B., Villalta, J.E., Chen, Y., Whitehead, E.H., Guimaraes, C., Panning, B., Ploegh, H.L., Bassik, M.C., et al. (2014). Genome-scale CRISPR-mediated control of gene repression and activation. Cell *159*, 647–661.

Hockemeyer, D., Soldner, F., Cook, E.G., Gao, Q., Mitalipova, M., and Jaenisch, R. (2008). A drug-inducible system for direct reprogramming of human somatic cells to pluripotency. Cell Stem Cell *3*, 346–353.

Horlbeck, M.A., Gilbert, L.A., Villalta, J.E., Adamson, B., Pak, R.A., Chen, Y., Fields, A.P., Park, C.Y., Corn, J.E., Kampmann, M., and Weissman, J.S. (2016). Compact and highly active next-generation libraries for CRISPR-mediated gene repression and activation. eLife *5*, e19760.

Kabadi, A.M., Ousterout, D.G., Hilton, I.B., and Gersbach, C.A. (2014). Multiplex CRISPR/Cas9-based genome engineering from a single lentiviral vector. Nucleic Acids Res. *42*, e147.

Keil, J.M., Qalieh, A., and Kwan, K.Y. (2018). Brain transcriptome databases: a user's guide. J. Neurosci. *38*, 2399–2412.

Klann, T.S., Black, J.B., and Gersbach, C.A. (2018). CRISPR-based methods for high-throughput annotation of regulatory DNA. Curr. Opin. Biotechnol. *52*, 32–41.

Konermann, S., Brigham, M.D., Trevino, A.E., Joung, J., Abudayyeh, O.O., Barcena, C., Hsu, P.D., Habib, N., Gootenberg, J.S., Nishimasu, H., et al. (2015). Genome-scale transcriptional activation by an engineered CRISPR-Cas9 complex. Nature *517*, 583–588.

Koopmans, F., van Nierop, P., Andres-Alonso, M., Byrnes, A., Cijsouw, T., Coba, M.P., Cornelisse, L.N., Farrell, R.J., Goldschmidt, H.L., Howrigan, D.P., et al. (2019). SynGO: an evidence-based, expert-curated knowledge base for the synapse. Neuron *103*, 217–234.e214.

Kreis, N.N., Louwen, F., and Yuan, J. (2019). The multifaceted p21 (Cip1/Waf1/*CDKN1A*) in cell differentiation, migration and cancer therapy. Cancers (Basel) *11*, 1220.

Kwon, J.B., Vankara, A., Ettyreddy, A.R., Bohning, J.D., and Gersbach, C.A. (2020). Myogenic progenitor cell lineage specification by CRISPR/Cas9-based transcriptional activators. Stem Cell Reports *14*, 755–769.

Lake, B.B., Chen, S., Sos, B.C., Fan, J., Kaeser, G.E., Yung, Y.C., Duong, T.E., Gao, D., Chun, J., Kharchenko, P.V., and Zhang, K. (2018). Integrative single-cell analysis of transcriptional and epigenetic states in the human adult brain. Nat. Biotechnol. *36*, 70–80.

Lambert, S.A., Jolma, A., Campitelli, L.F., Das, P.K., Yin, Y., Albu, M., Chen, X., Taipale, J., Hughes, T.R., and Weirauch, M.T. (2018). The human transcription factors. Cell *172*, 650–665.

Langmead, B., and Salzberg, S.L. (2012). Fast gapped-read alignment with Bowtie 2. Nat. Methods *9*, 357–359.

Lee, J., Sayed, N., Hunter, A., Au, K.F., Wong, W.H., Mocarski, E.S., Pera, R.R., Yakubov, E., and Cooke, J.P. (2012). Activation of innate immunity is required for efficient nuclear reprogramming. Cell *151*, 547–558.

Lee, J., Taylor, S.E.B., Smeriglio, P., Lai, J., Maloney, W.J., Yang, F., and Bhutani, N. (2015). Early induction of a prechondrogenic population allows efficient generation of stable chondrocytes from human induced pluripotent stem cells. FASEB J. *29*, 3399–3410.

Liu, P., Chen, M., Liu, Y., Qi, L.S., and Ding, S. (2018a). CRISPR-based chromatin remodeling of the endogenous Oct4 or Sox2 locus enables reprogramming to pluripotency. Cell Stem Cell *22*, 252–261.e254.

Liu, Y., Yu, C., Daley, T.P., Wang, F., Cao, W.S., Bhate, S., Lin, X., Still, C., 2nd, Liu, H., Zhao, D., et al. (2018b). CRISPR activation screens systematically identify factors that drive neuronal fate and reprogramming. Cell Stem Cell *23*, 758–771.e758.

Love, M.I., Huber, W., and Anders, S. (2014). Moderated estimation of fold change and dispersion for RNA-seq data with DESeq2. Genome Biol. *15*, 550.

Mertens, J., Marchetto, M.C., Bardy, C., and Gage, F.H. (2016). Evaluating cell reprogramming, differentiation and conversion technologies in neuroscience. Nat. Rev. Neurosci. *17*, 424–437.

Miller, J.A., Ding, S.L., Sunkin, S.M., Smith, K.A., Ng, L., Szafer, A., Ebbert, A., Riley, Z.L., Royall, J.J., Aiona, K., et al. (2014). Transcriptional landscape of the prenatal human brain. Nature *508*, 199–206.

Montalbano, A., Canver, M.C., and Sanjana, N.E. (2017). High-throughput approaches to pinpoint function within the noncoding genome. Mol. Cell *68*, 44–59.

Morris, S.A., Cahan, P., Li, H., Zhao, A.M., San Roman, A.K., Shivdasani, R.A., Collins, J.J., and Daley, G.Q. (2014). Dissecting engineered cell types and enhancing cell fate conversion via CellNet. Cell *158*, 889–902.

Najm, F.J., Strand, C., Donovan, K.F., Hegde, M., Sanson, K.R., Vaimberg, E.W., Sullender, M.E., Hartenian, E., Kalani, Z., Fusi, N., et al. (2018). Orthologous CRISPR-Cas9 enzymes for combinatorial genetic screens. Nat. Biotechnol. *36*, 179–189.

O'Geen, H., Bates, S.L., Carter, S.S., Nisson, K.A., Halmai, J., Fink, K.D., Rhie, S.K., Farnham, P.J., and Segal, D.J. (2019). Ezh2-dCas9 and KRAB-dCas9 enable engineering of epigenetic memory in a context-dependent manner. Epigenetics Chromatin *12*, 26.

Pang, Z.P., Yang, N., Vierbuchen, T., Ostermeier, A., Fuentes, D.R., Yang, T.Q., Citri, A., Sebastiano, V., Marro, S., Südhof, T.C., and Wernig, M. (2011). Induction of human neuronal cells by defined transcription factors. Nature *476*, 220–223.

Parekh, U., Wu, Y., Zhao, D., Worlikar, A., Shah, N., Zhang, K., and Mali, P. (2018). Mapping cellular reprogramming via pooled overexpression screens with paired fitness and single-cell RNA-sequencing readout. Cell Syst. *7*, 548–555.e8.

Rackham, O.J.L., Firas, J., Fang, H., Oates, M.E., Holmes, M.L., Knaupp, A.S., Suzuki, H., Nefzger, C.M., Daub, C.O., Shin, J.W., et al.; FANTOM Consortium (2016). A predictive computational framework for direct reprogramming between human cell types. Nat. Genet. *48*, 331–335.

Sagal, J., Zhan, X., Xu, J., Tilghman, J., Karuppagounder, S.S., Chen, L., Dawson, V.L., Dawson, T.M., Laterra, J., and Ying, M. (2014). Proneural transcription factor Atoh1 drives highly efficient differentiation of human pluripotent stem cells into dopaminergic neurons. Stem Cells Transl. Med. *3*, 888–898.

Salmon, P., and Trono, D. (2007). Production and titration of lentiviral vectors. Curr. Protoc. Hum. Genet. *Chapter 12*, Unit 12.10.

Sanson, K.R., Hanna, R.E., Hegde, M., Donovan, K.F., Strand, C., Sullender, M.E., Vaimberg, E.W., Goodale, A., Root, D.E., Piccioni, F., and Doench, J.G. (2018). Optimized libraries for CRISPR-Cas9 genetic screens with multiple modalities. Nat. Commun. *9*, 5416.

Shen, J.P., Zhao, D., Sasik, R., Luebeck, J., Birmingham, A., Bojorquez-Gomez, A., Licon, K., Klepper, K., Pekin, D., Beckett, A.N., et al. (2017). Combinatorial CRISPR-Cas9 screens for de novo mapping of genetic interactions. Nat. Methods *14*, 573–576.

Skene, N.G., Bryois, J., Bakken, T.E., Breen, G., Crowley, J.J., Gaspar, H.A., Giusti-Rodriguez, P., Hodge, R.D., Miller, J.A., Muñoz-Manchado, A.B., et al.; Major Depressive Disorder Working Group of the Psychiatric Genomics Consortium (2018). Genetic identification of brain cell types underlying schizophrenia. Nat. Genet. *50*, 825–833.

Takahashi, K., and Yamanaka, S. (2006). Induction of pluripotent stem cells from mouse embryonic and adult fibroblast cultures by defined factors. Cell *126*, 663–676.

Takahashi, K., and Yamanaka, S. (2016). A decade of transcription factor-mediated reprogramming to pluripotency. Nat. Rev. Mol. Cell Biol. *17*, 183–193.

Teratani-Ota, Y., Yamamizu, K., Piao, Y., Sharova, L., Amano, M., Yu, H., Schlessinger, D., Ko, M.S., and Sharov, A.A. (2016). Induction of specific neuron types by overexpression of single transcription factors. In Vitro Cell Dev. Biol. Anim. *52*, 961–973.

Thakore, P.I., Kwon, J.B., Nelson, C.E., Rouse, D.C., Gemberling, M.P., Oliver, M.L., and Gersbach, C.A. (2018). RNA-guided transcriptional silencing in vivo with S. aureus CRISPR-Cas9 repressors. Nat. Commun. *9*, 1674.

The Gene Ontology Consortium (2017). Expansion of the Gene Ontology knowledgebase and resources. Nucleic Acids Res. *45* (*D1*), D331–D338.

Theodorou, E., Dalembert, G., Heffelfinger, C., White, E., Weissman, S., Corcoran, L., and Snyder, M. (2009). A high throughput embryonic stem cell screen identifies Oct-2 as a bifunctional regulator of neuronal differentiation. Genes Dev. *23*, 575–588.

Tian, R., Gachechiladze, M.A., Ludwig, C.H., Laurie, M.T., Hong, J.Y., Nathaniel, D., Prabhu, A.V., Fernandopulle, M.S., Patel, R., Abshari, M., et al. (2019). CRISPR interference-based platform for multimodal genetic screens in human iPSC-derived neurons. Neuron *104*, 239–255.e212.

Tsunemoto, R., Lee, S., Szűcs, A., Chubukov, P., Sokolova, I., Blanchard, J.W., Eade, K.T., Bruggemann, J., Wu, C., Torkamani, A., et al. (2018). Diverse reprogramming codes for neuronal identity. Nature *557*, 375–380.

Vaquerizas, J.M., Kummerfeld, S.K., Teichmann, S.A., and Luscombe, N.M. (2009). A census of human transcription factors: function, expression and evolution. Nat. Rev. Genet. *10*, 252–263.

Vierbuchen, T., and Wernig, M. (2011). Direct lineage conversions: unnatural but useful? Nat. Biotechnol. *29*, 892–907.

Vierbuchen, T., and Wernig, M. (2012). Molecular roadblocks for cellular reprogramming. Mol. Cell *47*, 827–838.

Vierbuchen, T., Ostermeier, A., Pang, Z.P., Kokubu, Y., Südhof, T.C., and Wernig, M. (2010). Direct conversion of fibroblasts to functional neurons by defined factors. Nature *463*, 1035–1041.

Wapinski, O.L., Vierbuchen, T., Qu, K., Lee, Q.Y., Chanda, S., Fuentes, D.R., Giresi, P.G., Ng, Y.H., Marro, S., Neff, N.F., et al. (2013). Hierarchical mechanisms for direct reprogramming of fibroblasts to neurons. Cell *155*, 621–635.

Westendorp, B., Mokry, M., Groot Koerkamp, M.J., Holstege, F.C., Cuppen, E., and de Bruin, A. (2012). E2F7 represses a network of oscillating cell cycle genes to control S-phase progression. Nucleic Acids Res. *40*, 3511–3523.

Wylie, C.J., Hendricks, T.J., Zhang, B., Wang, L., Lu, P., Leahy, P., Fox, S., Maeno, H., and Deneris, E.S. (2010). Distinct transcriptomes define rostral and caudal serotonin neurons. J. Neurosci. *30*, 670–684.

Xu, S.J., and Heller, E.A. (2019). Recent advances in neuroepigenetic editing. Curr. Opin. Neurobiol. *59*, 26–33.

Xu, J., Du, Y., and Deng, H. (2015). Direct lineage reprogramming: strategies, mechanisms, and applications. Cell Stem Cell *16*, 119–134.

Xue, Y., Zhan, X., Sun, S., Karuppagounder, S.S., Xia, S., Dawson, V.L., Dawson, T.M., Laterra, J., Zhang, J., and Ying, M. (2019). Synthetic mRNAs drive highly efficient iPS cell differentiation to dopaminergic neurons. Stem Cells Transl. Med. *8*, 112–123.

Yang, N., Chanda, S., Marro, S., Ng, Y.H., Janas, J.A., Haag, D., Ang, C.E., Tang, Y., Flores, Q., Mall, M., et al. (2017). Generation of pure GABAergic neurons by transcription factor programming. Nat. Methods *14*, 621–628.

Zhang, Y., Pak, C., Han, Y., Ahlenius, H., Zhang, Z., Chanda, S., Marro, S., Patzke, C., Acuna, C., Covy, J., et al. (2013). Rapid single-step induction of functional neurons from human pluripotent stem cells. Neuron *78*, 785–798.

CellPress
OPEN ACCESS

STAR★METHODS

KEY RESOURCES TABLE

REAGENT or RESOURCE	SOURCE	IDENTIFIER
Antibodies		
Mouse monoclonal anti-TUBB3	Biolegend	Cat#: 801201; RRID: AB_2313773
Rabbit polyclonal anti-MAP2	Millipore Sigma	Cat#: AB5622; RRID: AB_91939
Mouse monoclonal anti-CD56 (NCAM)	ThermoFisher	Cat#: 12-0567; RRID: AB_10598200
Bacterial and Virus Strains		
Endura Electrocompetent Cells	Endura	Cat#: 60242
Chemicals, Peptides, and Recombinant Proteins		
Rock Inhibitor (Y-27632)	StemCell Tech	Cat#: 72304
Puromycin	Sigma	Cat#: P8833
Gentamicin	Sigma	Cat#: G1397
BsmBI	NEB	Cat#: R0739
Fetal Bovine Serum (FBS)	Sigma	Cat#: F2442
Penicillin-Streptomycin	ThermoFisher	Cat#: 15140122
Lenti-X Concentrator	Clontech	Cat#: 631232
Lipofectamine 3000	Invitrogen	Cat#: L3000008
Bovine Serum Albumin (BSA)	Sigma	Cat#: A7906
EDTA	Sigma	Cat#: E7889
Q5 High-Fidelity DNA Polymerase	NEB	Cat#: M0491
Agencourt AMPure XP SPRI beads	Beckman Coulter	Cat#: A63880
Doxycycline	Sigma	Cat#: D9891
DAPI	ThermoFisher	Cat#: D3571
BDNF	Peprotech	Cat#: 450-02
GDNF	Peprotech	Cat#: 450-01
NT-3	Peprotech	Cat#: 450-03
Critical Commercial Assays		
P3 Primary Cell 4D-Nucleofector kit	Lonza	Cat#: V4XP-3032
QuickExtract DNA Extraction Solution	Lucigen	Cat#: QE09050
DNeasy Blood and Tissue Kit	QIAGEN	Cat#: 69506
RNeasy Plus Mini Kit	QIAGEN	Cat#: 74136
Superscript VILO Reverse Transcription Kit	ThermFisher	Cat#: 11754
Perfecta SYBR Green Fastmix Kit	Quanta BioSciences	Cat#: 95072
Truseq Stranded mRNA Kit	Illumina	Cat#: 20020594
Deposited Data		
Pooled CRISPRa screens in *TUBB3-2A-mCherry* iPSCs	This paper	GEO: GSE159341
RNA-sequencing samples	This paper	GEO: GSE159341
Experimental Models: Cell Lines		
HEK293T	ATCC	Cat#: CRL-3216; RRID: CVCL_0063
H9 hESC (WA09)	WiCell	RRID: CVCL_9773
RVR-iPSC	Lee et al., 2012, 2015	N/A
Human Astrocyte	Lonza	Cat#: CC-2565
Oligonucleotides		
gRNA sequences: See Table S3	This paper	N/A
Primers for qRT-PCR: See Table S4	This paper	N/A

(Continued on next page)

Continued		
REAGENT or RESOURCE	SOURCE	IDENTIFIER
Primers used in Miseq: See Method Details	This paper	N/A
Recombinant DNA		
pLV_hUbC-dCas9-2xVP64-T2A-BSD	This paper	Addgene ID: 162333
pLV_hU6-sgRNA_hUbC-dSaCas9-KRAB-T2A-PuroR	This paper	Addgene ID: 162334
pLV_hU6-gRNA_hUbC-GFP-P2A-PuroR	This paper	Addgene ID: 162335
pLV_mU6-sgNGN3_hU6-gRNA_hUbC-GFP-P2A-PuroR	This paper	Addgene ID: 162336
pLV_mU6-sgASCL1_hU6-gRNA_hUbC-GFP-P2A-PuroR	This paper	Addgene ID: 162337
FUW-M2rtTA	Hockemeyer et al., 2008	Addgene ID: 20342
pTet-O-NEUROD1-T2A-PuroR	This paper	Addgene ID: 162338
pTet-O-NEUROG1-T2A-PuroR	This paper	Addgene ID: 162339
pTet-O-Ngn2-puro	Zhang et al., 2013	Addgene ID: 52047
pTet-O-NEUROG3-T2A-PuroR	This paper	Addgene ID: 162341
pTet-O-ATOH1-T2A-PuroR	This paper	Addgene ID: 162342
pTet-O-ATOH7-T2A-PuroR	This paper	Addgene ID: 162343
pTet-O-NR5A1-T2A-PuroR	This paper	Addgene ID: 162344
pTet-O-ASCL1-T2A-PuroR	This paper	Addgene ID: 162345
pTet-O-KLF7-T2A-PuroR	This paper	Addgene ID: 162346
pTet-O-OVOL1-T2A-PuroR	This paper	Addgene ID: 162347
pTet-O-E2F7-T2A-PuroR	This paper	Addgene ID: 162348
pTet-O-RUNX3-T2A-PuroR	This paper	Addgene ID: 162349
pTet-O-LHX8-T2A-PuroR	This paper	Addgene ID: 162350
Software and Algorithms		
Bowtie 2	Langmead and Salzberg, 2012	N/A
DESeq2	Love et al., 2014	N/A
STAR aligner	Dobin et al., 2013	N/A
http://geneontology.org	The Gene Ontology Consortium, 2017	N/A

RESOURCE AVAILABILITY

Lead Contact

Further information and requests for resources and reagents should be directed to and will be fulfilled by the Lead Contact, Charles A Gersbach (charles.gersbach@duke.edu)

Materials Availability

Plasmids generated in this study have been deposited to Addgene (Addgene ID #s 162333-162350).

Data and Code Availability

Raw and processed data for the RNA-sequencing and gRNA library sequencing generated in this study have been deposited in the NCBI Gene Expression Omnibus under accession number GSE159341.

EXPERIMENTAL MODEL AND SUBJECT DETAILS

Cell Culture

Human iPSCs and ESCs were maintained on matrigel (Corning, 354230) dishes in mTesR (Stemcell Tech, 85850). For neuronal differentiation experiments, the medium was changed to neurogenic medium (DMEM/F-12 Nutrient Mix (GIBCO, 11320), 1x B-27 serum-free supplement (GIBCO, 17504), 1x N-2 supplement (GIBCO, 17502), and 25 µg/mL gentamicin (Sigma, G1397)). Human astrocytes (Lonza, CC-2565) were maintained in DMEM High Glucose supplemented with 10% FBS (Sigma, F2442) and 1% penicillin-streptomycin (GIBCO, 15140122) and transferred to neurogenic medium for co-culture with iPSC-derived neurons. For lentivirus production, HEK293T cells were cultured in DMEM High Glucose supplemented with 10% FBS and 1% penicillin-streptomycin.

Construction of a TUBB3-2A-mCherry pluripotent stem cell line

A human iPS cell line (RVR-iPSCs) was used to construct the *TUBB3-2A-mCherry* reporter line. RVR-iPSCs were retrovirally reprogrammed from BJ fibroblasts and characterized previously (Lee et al., 2012, 2015). To generate the *TUBB3-2A-mCherry* reporter line, 3×10^6 cells were dissociated with Accutase (Stemcell Tech, 7920) and electroporated with 6 μg of gRNA-Cas9 expression vector and 3 μg of *TUBB3* targeting vector using the P3 Primary Cell 4D-Nucleofector Kit (Lonza, V4XP-3032). Transfected cells were plated into a 10 cm dish coated with Matrigel (Corning, 354230) in compete mTesR (Stemcell Tech, 85850) supplemented with 10 μM Rock Inhibitor (Y-27632, Stemcell Tech, 72304). 24 hours after transfection, positive selection began with 1 μg/mL puromycin for 7 days. Following selection, cells were transfected with a CMV-CRE recombinase expression vector to remove the floxed puromycin selection cassette. Transfected cells were expanded and plated at low density for clonal isolation (180 cells/cm^2). Resulting clones were mechanically picked and expanded and gDNA was extracted using QuickExtract DNA Extraction Solution (Lucigen, QE09050) for PCR screening of targeting vector integration. A second round of clonal isolation was performed using the same protocol following lentiviral transduction of ^{VP64}dCas9^{VP64}.

METHOD DETAILS

Plasmid construction

The lentiviral ^{VP64}dCas9^{VP64} plasmid was generated by modifying Addgene plasmid #59791 to replace GFP with the BSD blasticidin resistance gene. The lentiviral dSaCas9^{KRAB} plasmid was generated by modifying Addgene plasmid #106249 to insert a *S. aureus* gRNA cassette with a *ZFP36L1, HES3* or scrambled non-targeting gRNA. The gRNA expression plasmid for the single CAS-TF screen was generated by modifying Addgene plasmid #83925 to contain an optimized gRNA scaffold (Chen et al., 2013) and a puromycin resistance gene in place of Bsr. The gRNA expression plasmids for the paired CAS-TF screens were generated by further modification of the single gRNA expression plasmid to contain an additional gRNA cassette expressing either sgNGN3 or sgASCL1 under control of the mU6 Pol III promoter with a modified gRNA scaffold described previously (Adamson et al., 2016). Individual gRNAs were ordered as oligonucleotides (Integrated DNA Technologies), phosphorylated, hybridized, and cloned into the gRNA expression plasmids using BsmBI sites. Protospacers used for individual gRNA cloning are listed in Table S3.

The *TUBB3* targeting vector was cloned by inserting ~700 bp homology arms (surrounding the *TUBB3* stop codon), amplified by PCR from genomic DNA of RVR-iPS cells, surrounding a P2A–mCherry sequence with a floxed puromycin resistance cassette.

cDNAs encoding TFs were either PCR amplified from cDNA pools or synthesized as gBlocks (Integrative DNA Technologies) and cloned into Addgene plasmid #52047 using EcoRI and XbaI restriction sites. TetO gene expression was achieved by co-delivery of M2rtTA (Addgene #20342). All new plasmids from this study are available via Addgene (Addgene ID #s 162333-162350).

Lentiviral production and titration

HEK293T cells were acquired from the American Tissue Collection Center (ATCC) and purchased through the Duke University Cell Culture Facility. The cells were maintained in DMEM High Glucose supplemented with 10% FBS and 1% penicillin-streptomycin and cultured at 37°C with 5% CO2. For lentiviral production of the gRNA libraries, ^{VP64}dCas9^{VP64} and dSaCas9^{KRAB}, 4.5×10^6 cells were transfected using the calcium phosphate precipitation method (Salmon and Trono, 2007) with 6 μg pMD2.G (Addgene #12259), 15 μg psPAX2 (Addgene #12260) and 20 μg of the transfer vector. The medium was exchanged 12-14 hours after transfection, and the viral supernatant was harvested 24 and 48 hours after this medium change. The viral supernatant was pooled and centrifuged at 600*g* for 10 min, passed through a PVDF 0.45 μm filter (Millipore, SLHV033RB) and concentrated to 50x in 1x PBS using Lenti-X Concentrator (Clontech, 631232) in accordance with the manufacturer's protocol.

To produce lentivirus for gRNA and cDNA validations, 0.4×10^6 cells were transfected using Lipofectamine 3000 (Invitrogen, L3000008) according to the manufacturer's instructions with 200 ng pMD2.G, 600 ng psPAX2, and 200 ng of the transfer vector. The medium was exchanged 12-14 hours after transfection, and the viral supernatant was harvested 24 and 48 hours after this medium change. The viral supernatant was pooled and centrifuged at 600*g* for 10 min and concentrated to 50x in 1x PBS using Lenti-X Concentrator (Clontech, 631232) in accordance with the manufacturer's protocol.

The titer of the lentiviral gRNA library pools for the single or paired CAS-TF libraries was determined by transducing 6×10^4 cells with serial dilutions of lentivirus and measuring the percent GFP expression 4 days after transduction with an Accuri C6 flow cytometer (BD). All lentiviral titrations were performed in the *TUBB3-2A-mCherry* cell line used in the CAS-TF single and paired gRNA screens.

CAS-TF gRNA library design and cloning

Putative TFs were selected from a previous catalog of human transcription factors (Vaquerizas et al., 2009). A gRNA library consisting of 5 gRNAs per TSS targeting 1,496 TFs was extracted from a previous genome-wide CRISPRa library (Horlbeck et al., 2016). The library included a set of 100 scrambled non-targeting gRNAs extracted from the same genome-wide library for a total of 8,435 gRNAs. The oligonucleotide pool (Custom Array) was PCR amplified and cloned using Gibson assembly into the single gRNA expression plasmid for the single CAS-TF screen or the dual gRNA expression plasmid for the paired CAS-TF screens with sgASCL1 or sgNGN3. The list of TFs and corresponding gRNA library is available for download in Table S5.

The sub-library was designed by extracting additional gRNAs from several previously published CRISPRa genome-wide libraries (Gilbert et al., 2014; Horlbeck et al., 2016; Konermann et al., 2015; Sanson et al., 2018) to obtain an average of 33 gRNAs per gene targeting 109 TFs. The library included a set of 300 scrambled non-targeting gRNAs for a total of 3,874 gRNAs. The oligonucleotide pool (Twist Bioscience) was PCR amplified and cloned into the single gRNA expression plasmid as done with the original CAS-TF library. The CAS-TF sub-library is available for download in Table S5.

Single and paired CAS-TF neuronal differentiation screens

Each CAS-TF screen was performed in triplicate with independent transductions. For each replicate, 24×10^6 *TUBB3-2A-mCherry* ^{VP64}dCas9^{VP64} iPSCs were dissociated using Accutase (Stemcell Tech, 7920) and transduced in suspension across five matrigel-coated 15-cm dishes in mTesR (Stemcell Tech 85850) supplemented with 10 μM Rock Inhibitor (Y-27632, Stemcell Tech, 72304). Cells were transduced at a MOI of 0.2 to obtain one gRNA per cell and ~550-fold coverage of the CAS-TF gRNA library. The medium was changed to fresh mTesR without Rock Inhibitor 18-20 hours after transduction. Antibiotic selection was started 30 hours after transduction by adding 1 μg/mL puromycin (Sigma, P8833) directly to the plates without changing the medium. 48 hours after transduction the medium was changed to neurogenic medium (DMEM/F-12 Nutrient Mix (GIBCO, 11320), 1x B-27 serum-free supplement (GIBCO, 17504), 1x N-2 supplement (GIBCO, 17502), and 25 μg/mL gentamicin (Sigma, G1397)) supplemented with 1 μg/mL puromycin for the remainder of the experiment with daily medium changes.

Cells were harvested for sorting 5 days after transduction of the gRNA library for the single factor CAS-TF screen and the sgASCL1 paired screen. Cells were harvested 4 days after transduction for the sgNGN3 paired screen. Cells were washed once with 1x PBS, dissociated using Accutase, filtered through a 30 μm CellTrics filter (Sysmex, 04-004-2326) and resuspended in FACS Buffer (0.5% BSA (Sigma, A7906), 2 mM EDTA (Sigma, E7889) in PBS). Before sorting, an aliquot of 4.8×10^6 cells was taken to represent a bulk unsorted population. The highest and lowest 5% of cells were sorted based on mCherry expression and 4.8×10^6 cells were sorted into each bin. Sorting was done with a SH800 FACS Cell Sorter (Sony Biotechnology). After sorting, genomic DNA was harvested with the DNeasy Blood and Tissue Kit (QIAGEN, 69506).

Sub-library screen

The CAS-TF sub-library screen was performed in triplicate with independent transductions. For each replicate, 9.6×10^6 *TUBB3-2A-mCherry* ^{VP64}dCas9^{VP64} iPSCs were dissociated using Accutase (Stemcell Tech, 7920) and transduced in suspension across two matrigel-coated 15-cm dishes in mTesR (Stemcell Tech 85850) supplemented with 10 μM Rock Inhibitor (Y-27632, Stemcell Tech, 72304). Cells were transduced at a MOI of 0.2 to obtain one gRNA per cell and ~495-fold coverage of the CAS-TF gRNA sub-library. The medium was changed to fresh mTesR without Rock Inhibitor 18-20 hours after transduction. Antibiotic selection was started 30 hours after transduction by adding 1 μg/mL puromycin (Sigma, P8833) directly to the plates without changing the medium. 48 hours after transduction the medium was changed to neurogenic medium (DMEM/F-12 Nutrient Mix (GIBCO, 11320), 1x B-27 serum-free supplement (GIBCO, 17504), 1x N-2 supplement (GIBCO, 17502), and 25 μg/mL gentamicin (Sigma, G1397)) supplemented with 1 μg/mL puromycin for the remainder of the experiment with daily medium changes.

Cells were harvested for sorting 5 days after transduction of the gRNA library. Cells were washed once with 1x PBS, dissociated using Accutase, filtered through a 30 μm CellTrics filter (Sysmex, 04-004-2326) and resuspended in FACS Buffer (0.5% BSA (Sigma, A7906), 2 mM EDTA (Sigma, E7889) in PBS). Before sorting, an aliquot of 2×10^6 cells was taken to represent a bulk unsorted population. The highest and lowest 5% of cells were sorted based on mCherry expression and 2×10^6 cells were sorted into each bin. Sorting was done with a SH800 FACS Cell Sorter (Sony Biotechnology). After sorting, genomic DNA was harvested with the DNeasy Blood and Tissue Kit (QIAGEN, 69506).

gRNA library sequencing

The gRNA libraries were amplified from each genomic DNA sample across 100 μL PCR reactions using Q5 hot start polymerase (NEB, M0493) with 1 μg of genomic DNA per reaction. The PCR amplification was done according to the manufacturer's instructions, using 25 cycles at an annealing temperature of 60°C with the following primers:

Fwd: 5′-AATGATACGGCGACCACCGAGATCTACACAATTTCTTGGGTAGTTTGCAGTT
Rev: 5′-CAAGCAGAAGACGGCATACGAGAT-(6-bp index sequence)- GACTCGGTGCCACTTTTTCAA

The amplified libraries were purified with Agencourt AMPure XP beads (Beckman Coulter, A63881) using double size selection of 0.65 × and then 1 × the original volume to purify the 282 bp amplicon. Each sample was quantified after purification with the Qubit dsDNA High Sensitivity assay kit (Thermo Fisher, Q32854). Samples were pooled and sequenced on a MiSeq (Illumina) with 20-bp paired-end sequencing using the following custom read and index primers:

Read1: 5′-GATTTCTTGGCTTTATATATCTTGTGGAAAGGACGAAACACCG
Index: 5′-GCTAGTCCGTTATCAACTTGAAAAAGTGGCACCGAGTC
Read2: 5′- GTTGATAACGGACTAGCCTTATTTAAACTTGCTATGCTGTTTCCAGCATAGCTCTTAAAC

In vivo expression comparison

RNA-sequencing data generated as part of the Brainspan Developmental Transcriptome Atlas was downloaded (Miller et al., 2014). The average expression for the 17 TFs identified in the single-factor CAS-TF screen was calculated for each developmental time point and anatomical region listed between 8 and 13 post conception weeks. A random set of 17 TFs was identically analyzed, and a representative comparison is shown in Figure 1F.

gRNA and cDNA validations

The top enriched gRNAs from the screens were cloned into the appropriate gRNA expression vector as described previously. The gRNA validations were performed similarly as done with the screens, except the transductions were performed in 24-well plates and the virus was delivered at high MOI. Cells were harvested for flow cytometry or qRT-PCR 4 days after gRNA transduction.

For immunofluorescence staining experiments, the cDNAs encoding the top enriched TFs were PCR amplified and cloned into a doxycycline inducible expression vector as described previously. Cells were co-transduced in suspension with the indicated TFs along with a separate lentivirus encoding the M2rtTA (Addgene #20342) in mTesR supplemented with 10 μM Rock Inhibitor. Unmodified iPSCs were used for these experiments to enable staining with red fluorophores without interference from the mCherry reporter. 18-20 hours after transduction, the medium was changed to neurogenic medium supplemented with 0.1 μg/mL doxycycline (Sigma, D9891). Staining was done 4 days after transduction as described previously. For a subset of the TFs, the *TUBB3-2A-mCherry* cell line was used to sort off the highest mCherry expressing cells 3 days after transduction. The cells were replated onto a pre-established monolayer of human astrocytes (Lonza, CC-2565) and cultured for an additional 8 days in neurogenic medium before staining. gRNA and cDNA validations in H9 human embryonic stem cells were performed similarly to those described for iPSCs. A polyclonal ^{VP64}dCas9^{VP64} H9 ESC line was established via lentiviral transductions, and gRNAs were delivered with a separate lentivirus.

Quantitative RT-PCR

Cells were dissociated with Accutase (StemCell Tech, 7920) and centrifuged at 300*g* for 5 min. Total RNA was isolated using RNeasy Plus (QIAGEN, 74136) and QIAshredder kits (QIAGEN, 79656). Reverse transcription was carried out on 0.1 μg total RNA per sample in a 10 μL reaction using the SuperScript VILO Reverse Transcription Kit (Invitrogen, 11754). 1.0 μL of cDNA was used per PCR reaction with Perfecta SYBR Green Fastmix (Quanta BioSciences, 95072) using the CFX96 Real-Time PCR Detection System (Bio-Rad). The amplification efficiencies over the appropriate dynamic range of all primers were optimized using dilutions of purified amplicon. All amplicon products were verified by gel electrophoresis and melting curve analysis. All qRT-PCR results are presented as fold change in RNA normalized to *GAPDH* expression. Primers used in this study can be found in Table S4.

Immunofluorescence staining

Cells were washed briefly with PBS and then fixed with 4% paraformaldehyde (Santa Cruz, sc-281692) for 20 minutes at room temperature. Cells were washed twice with PBS and then incubated with blocking buffer (10% goat serum (Sigma, G6767), 2% BSA (Sigma, A7906) in PBS) for 30 min at room temperature. Cells were permeabilized with 0.2% Triton X-100 (Sigma, T8787) for 10 min at room temperature. The following primary antibodies were used with incubations for 2 hours at room temperature: Mouse anti-TUBB3 (1:1000 dilution, BioLegend, 801201); Rabbit anti-MAP2 (1:500 dilution, Sigma, AB5622). Cells were washed three times with PBS and then incubated with secondary antibody and DAPI (Invitrogen, D3571) in blocking solution for 1 hour at room temperature. The following secondary antibodies were used: Alexa Fluor 488 goat anti-mouse (1:500 dilution, Invitrogen, A-11001); Alexa Fluor 594 goat anti-rabbit (1:500 dilution, Invitrogen, A-11012). Cells were washed three times with PBS and imaged with a Zeiss 780 upright confocal microscope.

For NCAM staining of live cells for gRNA validations, cells were dissociated with Accutase (Stemcell Tech, 7920), centrifuged at 300*g* for 5 min, and resuspended in staining buffer (0.5% BSA (Sigma, A7906) and 2 mM EDTA (Sigma, E7889) in PBS) at 10×10^6 cells per mL. Mouse anti-CD56 (NCAM, Invitrogen, 12-0567) was added at 0.6 μg per 1×10^6 cells and incubated for 30 min at 4°C. Cells were washed with 1 mL staining buffer, centrifuged at 300*g* for 5 min and resuspended in staining buffer for analysis on the SH800 FACS Cell Sorter (Sony Biotechnology).

RNA-sequencing with tetO cDNA expression

TUBB3-2A-mCherry iPSCs were co-transduced with a lentivirus encoding M2rtTA and the indicated tetO-cDNA. Cells were transduced in mTesR with 10 μM Rock Inhibitor. The following day, the medium was changed to neurogenic medium (DMEM/F-12 Nutrient Mix (GIBCO, 11320), 1x B-27 serum-free supplement (GIBCO, 17504), 1x N-2 supplement (GIBCO, 17502), and 25 μg/mL gentamicin (Sigma, G1397)) supplemented with 0.1 μg/mL doxycycline. Cells were sorted after 2 or 3 days of transgene expression using a SH800 FACS Cell Sorter in semi-purity mode. Sorted cells were replated onto matrigel-coated 24-well plates and cultured in neurogenic medium supplemented with 10 ng/mL each of BDNF, GDNF and NT-3 (PeproTech) until harvest after 6 or 7 days.

Total RNA was extracted using RNeasy Mini Kit (QIAGEN) and 100 ng of RNA was used to develop RNA-seq libraries. RNA-sequencing libraries were prepared using the Truseq Stranded mRNA kit (Illumina) according to the manufacturer's protocol. The libraries were sequenced on a NextSeq 500 on High Output Mode with 75 bp paired-end reads.

Electrophysiology

TUBB3-2A-mCherry iPSCs were co-transduced with a lentivirus encoding M2rtTA and either tetO-NEUROG3 alone or in combination with tetO-LHX8. Cells were transduced in mTesR with 10 μM Rock Inhibitor. The following day, the medium was changed to neurogenic medium supplemented with 0.1 μg/mL doxycycline. Cells were sorted after 3 days of transgene expression using a SH800 FACS Cell Sorter in semi-purity mode. Sorted cells were replated onto matrigel-coated coverslips and cultured in neurogenic medium supplemented with 10 ng/mL each of BDNF, GDNF and NT-3 (PeproTech) for the remainder of the experiment.

Whole-cell patch-clamp recordings were performed on cultured cells 7 days post-induction of transgene expression under a Zeiss Axio Examiner.D1 microscope. To avoid osmotic shock, culture media was gradually changed to artificial CSF (aCSF) in a stepwise manner over approximately 5 minutes, and then the coverslip was moved to the recording chamber. aCSF contained 124mM NaCl, 26mM $NaHCO_3$, 10mM D-glucose, 2mM $CaCl_2$, 3mM KCl, 1.3mM $MgSO_4$, and 1.25mM NaH_2PO_4 (310 mOsm/L), and was continuously bubbled at room temperature with 95% O_2 and 5% CO_2. Cells were inspected under a 20x water-immersion objective using infrared illumination and differential interference contrast optics (IR-DIC). The experimenter was blinded to the condition and chose the most morphologically complex neurons for recording. Electrodes (4-7 MΩ) were pulled from borosilicate glass capillaries using a P-97 puller (Sutter Instrument) and filled with an intracellular solution containing 135mM K-methanesulfonate, 8mM NaCl, 10mM HEPES, 0.3mM EGTA, 4mM MgATP, and 0.3mM Na_2GTP (pH 7.3 with KOH, adjusted to 295 mOsm/L with sucrose). After gigaohm seals were ruptured, membrane resistance was measured in voltage-clamp mode with a brief hyperpolarizing pulse, and membrane capacitance was estimated from the capacitance compensation circuitry of the amplifier. Then, resting membrane potential was recorded in current-clamp mode. Finally, a small holding current was applied to adjust the membrane potential to around −60mV, and input-output curves were generated by injecting increasing amounts of current. Data were recorded with a Multiclamp 700B amplifier (Molecular Devices) and digitized at 50kHz with a Digidata 1550 (Molecular Devices). Action potential properties were calculated based on the first action potential generated using custom MATLAB scripts. Action potentials were counted by visual inspection if they had the characteristic two-component rising phase, regardless of peak amplitude. All experiments were analyzed blinded to the condition, and only recordings which remained stable over the entire period of data collection were used.

Orthogonal CRISPR-based gene regulation

TUBB3-2A-mCherry ^{VP64}dCas9^{VP64} iPSCs were transduced with an all-in-one dSaCas9^{KRAB} lentivirus (Thakore et al., 2018) containing either a *ZFP36L1*, *HES3* or scrambled *S. aureus* gRNA. After 2 days, antibiotic selection was started with 0.5 μg/mL puromycin and cells were cultured for an additional 7 days in mTesR. After 9 days following transduction with dSaCas9^{KRAB} and *S. aureus* gRNAs, cells were transduced with a lentivirus encoding either sgNGN3 or sgASCL1 and switched to neurogenic medium. Cells were harvested 3 days after gRNA transduction for mRNA-sequencing and 4 days after gRNA transduction for flow cytometry.

Total RNA was isolated using RNeasy Plus (QIAGEN, 74136) and QIAshredder kits (QIAGEN, 79656). Libraries were prepped and sequenced by Genewiz on an Illumina Hiseq with 2x150 bp paired-end reads. The mean quality score for the sequencing run was 39.03 with 94.48% reads ≥ 30. The average number of reads per sample was ~50M reads. mRNA-sequencing analysis was done as described previously for the tetO cDNA experiments. GFP transgene expression was quantified using bowtie2 to align trimmed reads to a custom GFP index generated with the bowtie2-build function. Raw counts were normalized for sequencing depth and displayed as relative counts across the three conditions analyzed.

QUANTIFICATION AND STATISTICAL ANALYSIS

Data processing and enrichment analysis for CRISPRa screens

FASTQ files were aligned to custom indexes of the 8,435 protospacers (generated from the bowtie2-build function) using Bowtie 2 (Langmead and Salzberg, 2012). Counts for each gRNA were extracted and used for further analysis. All enrichment analysis was done with R. Individual gRNA enrichment was determined using the DESeq2 (Love et al., 2014) package to compare gRNA abundance between high and low, unsorted and low, or unsorted and high conditions for each screen. TFs were selected as hits if two or more gRNAs were significantly enriched (FDR < 0.01) in the mCherry-high cell bin relative to both the unsorted and the mCherry-low cell bins. Table S5 includes raw counts and corresponding DESeq2 differential expression results for each screen performed in this study.

RNA-sequencing analysis

Reads were first trimmed using Trimmomatic v0.32 to remove adapters and then aligned to GRCh38 using STAR aligner (Dobin et al., 2013). Gene counts were obtained with featureCounts from the subread package (version 1.4.6-p4) using the comprehensive gene annotation in Gencode v22. Differential expression analysis was determined with DESeq2 where gene counts are fitted into negative binomial generalized linear models (GLMs) and Wald statistics determine significant hits. Genes were included for analysis if at least three samples across all conditions tested had a TPM > 1. Gene Ontology analyses were performed using the Gene Ontology Consortium database (2017) and Synaptic Gene Ontology Consortium database (Koopmans et al., 2019).

Statistical methods

Statistical analysis was done using GraphPad Prism 7. See figure legends for details on specific statistical tests run for each experiment. Statistical significance is represented by a star (*) and indicates a computed p value < 0.05.

Cell Reports, Volume *33*

Supplemental Information

Master Regulators and Cofactors of Human Neuronal Cell Fate Specification Identified by CRISPR Gene Activation Screens

Joshua B. Black, Sean R. McCutcheon, Shataakshi Dube, Alejandro Barrera, Tyler S. Klann, Grayson A. Rice, Shaunak S. Adkar, Scott H. Soderling, Timothy E. Reddy, and Charles A. Gersbach

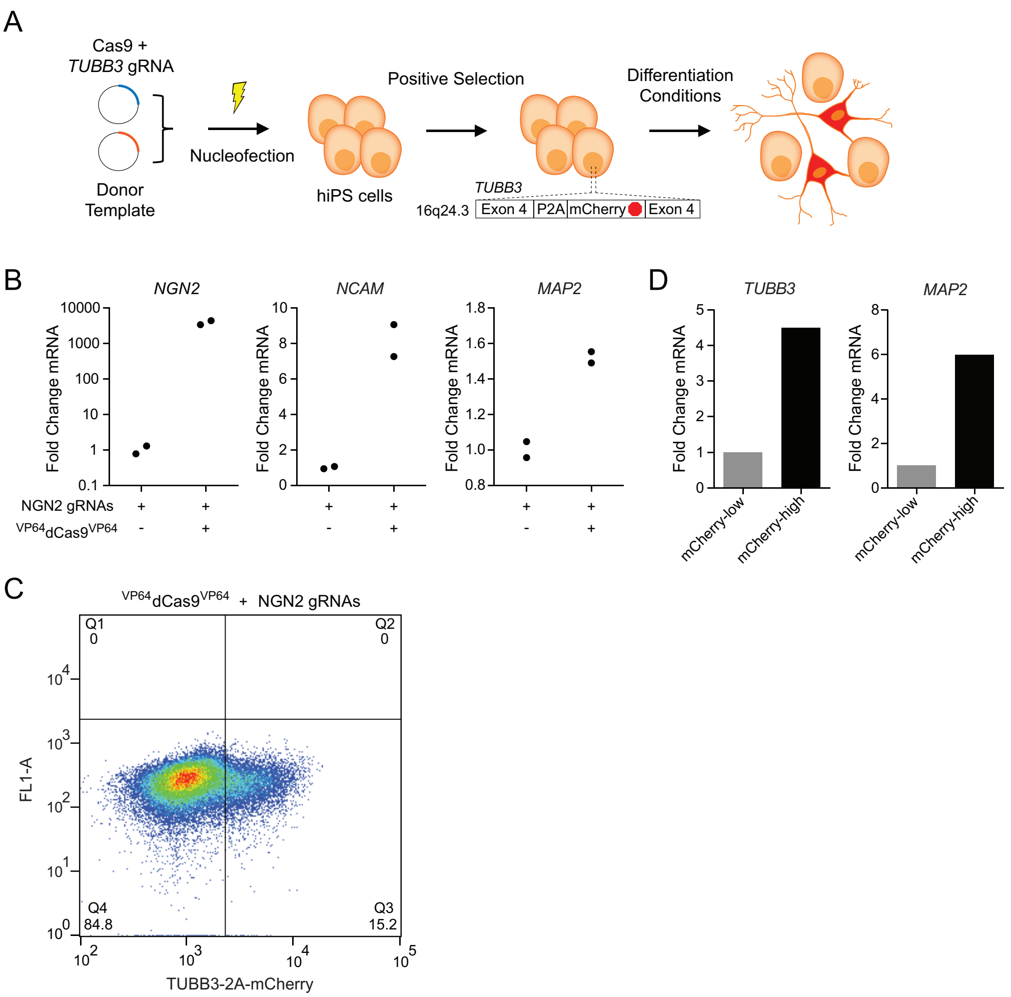

Figure S1. Generation and characterization of a *TUBB3-2A-mCherry* reporter cell line. (A) Schematic representation of the knock-in of a *P2A-mCherry* cassette into exon four of *TUBB3* in a human pluripotent stem cell line using Cas9 nuclease and a donor template. (B) Targeted activation of endogenous *NEUROG2* in pluripotent stem cells with ^{VP64}dCas9^{VP64} and a set of four gRNAs targeting the *NEUROG2* promoter. Expression of *NCAM* (middle) and *MAP2* (right) with targeted activation of *NEUROG2* (n = 2 biological replicates). (C) TUBB3-2A-mCherry expression by flow cytometry with targeted activation of *NEUROG2* with ^{VP64}dCas9^{VP64} and a set of four gRNAs targeting the promoter. (D) *TUBB3* and *MAP2* expression in *TUBB3-2A-mCherry* cells sorted for the highest and lowest mCherry expression after activation of *NEUROG2* with ^{VP64}dCas9^{VP64} and gRNAs (n = 1 biological replicate). Related to Figure 1.

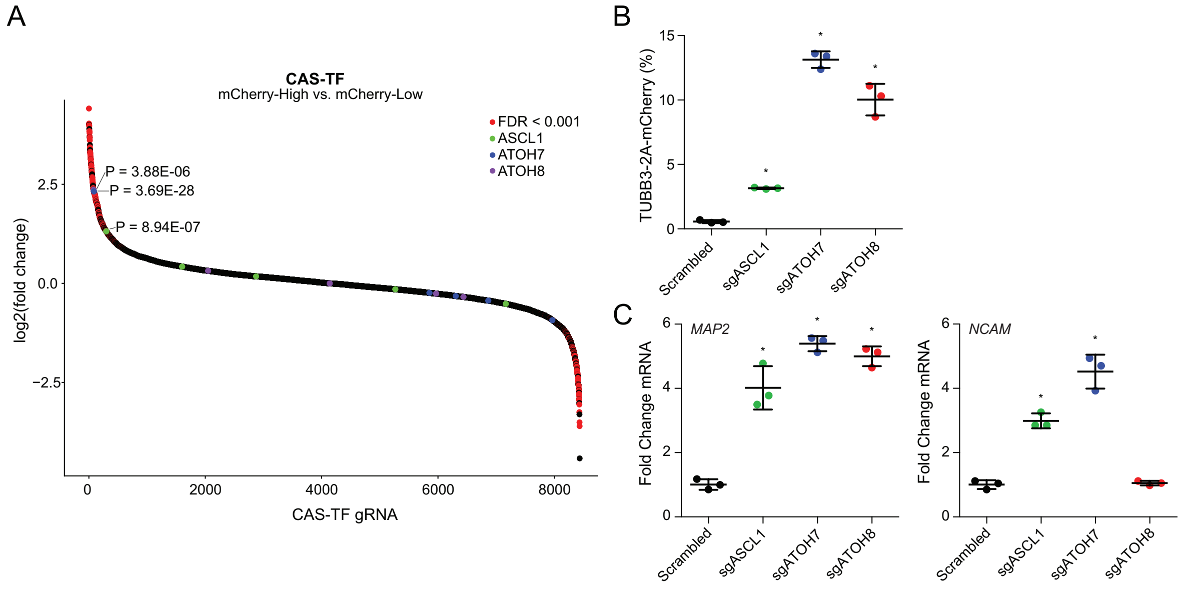

Figure S2. Validations of TFs with a single enriched gRNA. (A) A ranked list of fold change in gRNA abundance between mCherry-High versus mCherry-Low expressing cells in the single factor CAS-TF screen. *ASCL1*, *ATOH7* and *ATOH8* all have a single gRNA significantly enriched. (B) Individual validations of sgASCL1, sgATOH7 and sgATOH8 for (B) percent TUBB3-2A-mCherry expression and (C) *MAP2* (left) and *NCAM* (right) expression four days after gRNA transduction (*$p < 0.05$ by global one-way ANOVA with Dunnett's post hoc test comparing all groups to a scrambled gRNA, n = 3 biological replicates, error bars represent SEM). Related to Figures 1 and 2.

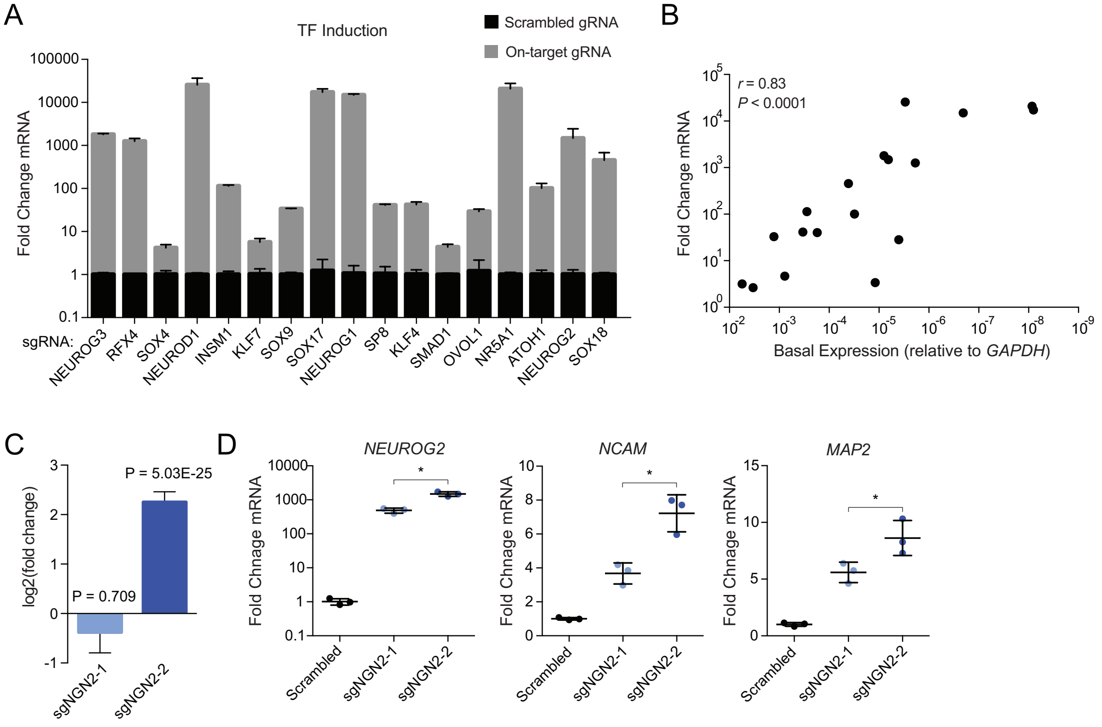

Figure S3. Endogenous induction of TFs with ^{VP64}dCas9^{VP64}. (A) Fold induction of a subset of 17 TFs enriched in the single factor CAS-TF screen with ^{VP64}dCas9^{VP64} and the top enriched gRNA (fold change relative to a scrambled gRNA, n = 2 biological replicates). (B) Relation between the fold induction of each TF and the basal expression of that TF relative to *GAPDH* expression. (C) Comparison of gRNA enrichment from the single factor CAS-TF screen for two *NEUROG2* gRNAs. (D) Validation of these two *NEUROG2* gRNAs for TF induction and expression of downstream neuronal markers (*p < 0.05 by global one-way ANOVA with a Tukey post hoc test comparing the two *NEUROG2* gRNAs, n = 3 biological replicates, error bars represent SEM). Related to Figures 1 and 2.

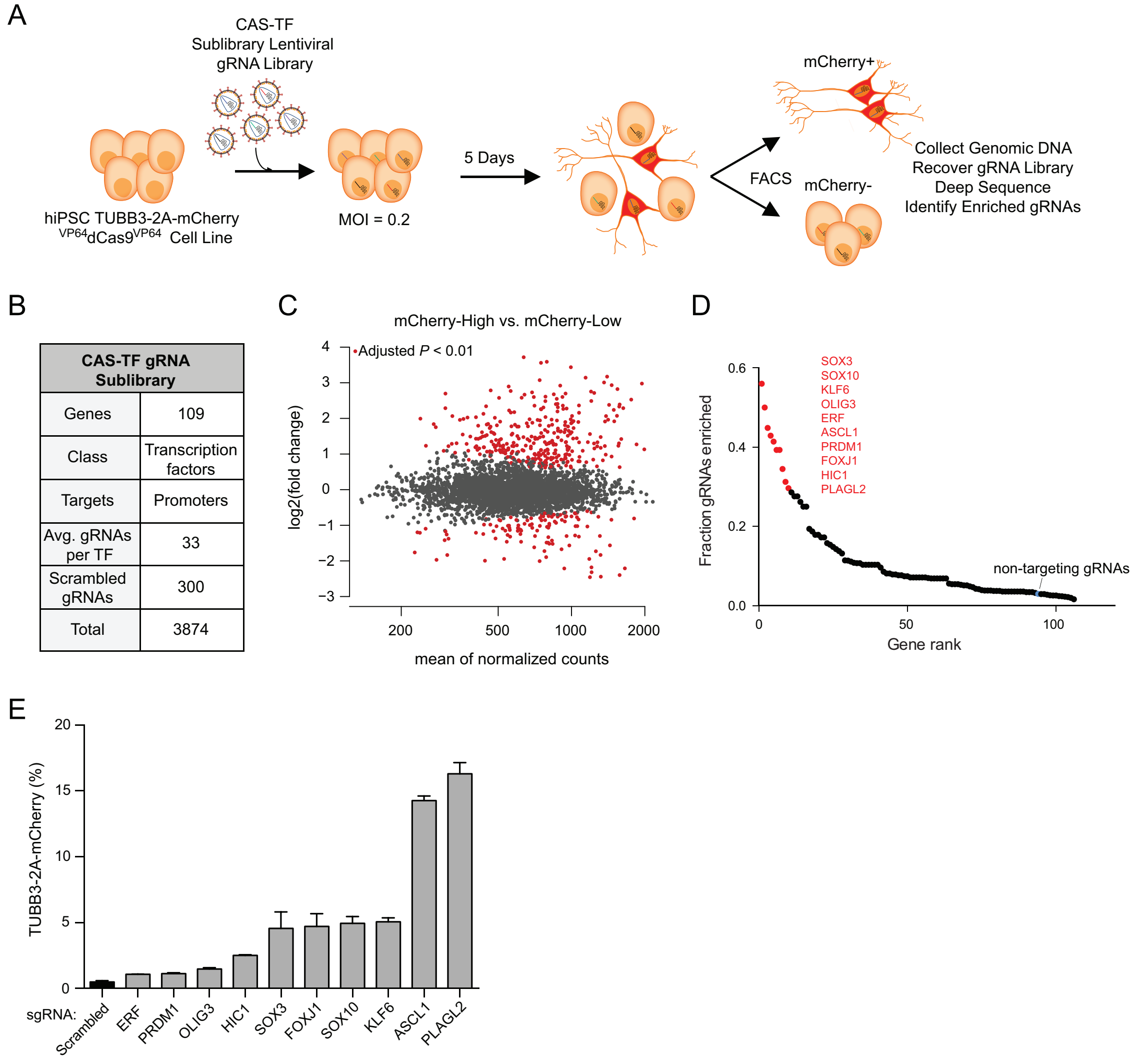

Figure S4. CAS-TF sub-library gRNA screen. (A) Schematic representation of the CRISPRa sub-library screen for neuronal-fate determining transcription factors in human pluripotent stem cells. A ^{VP64}dCas9^{VP64} *TUBB3-2A-mCherry* reporter cell line was transduced with the CAS-TF pooled lentiviral library at an MOI of 0.2 and sorted for mCherry expression via FACS. gRNA abundance in each cell bin was measured by deep sequencing, and depleted or enriched gRNAs were identified by differential expression analysis. (B) The CAS-TF gRNA sub-library was extracted from several previous genome-wide CRISPRa library (Gilbert et al., 2014; Horlbeck, 2016; Konermann et al., 2015; Sanson et al., 2018) and consists of 3,874 gRNAs targeting 109 putative transcription factors (~33 gRNAs per gene). (C) Differential expression analysis of normalized gRNA counts between the mCherry-High and mCherry-Low cell populations. Red data points indicate FDR < 0.01 by differential DESeq2 analysis (n = 3 biological replicates). (D) Ranked list of percent enriched gRNAs per gene. (E) Validations of 10 factors for TUBB3-2A-mCherry expression four days after transduction of gRNAs (n = 2 biological replicates). Related to Figures 1 and 2.

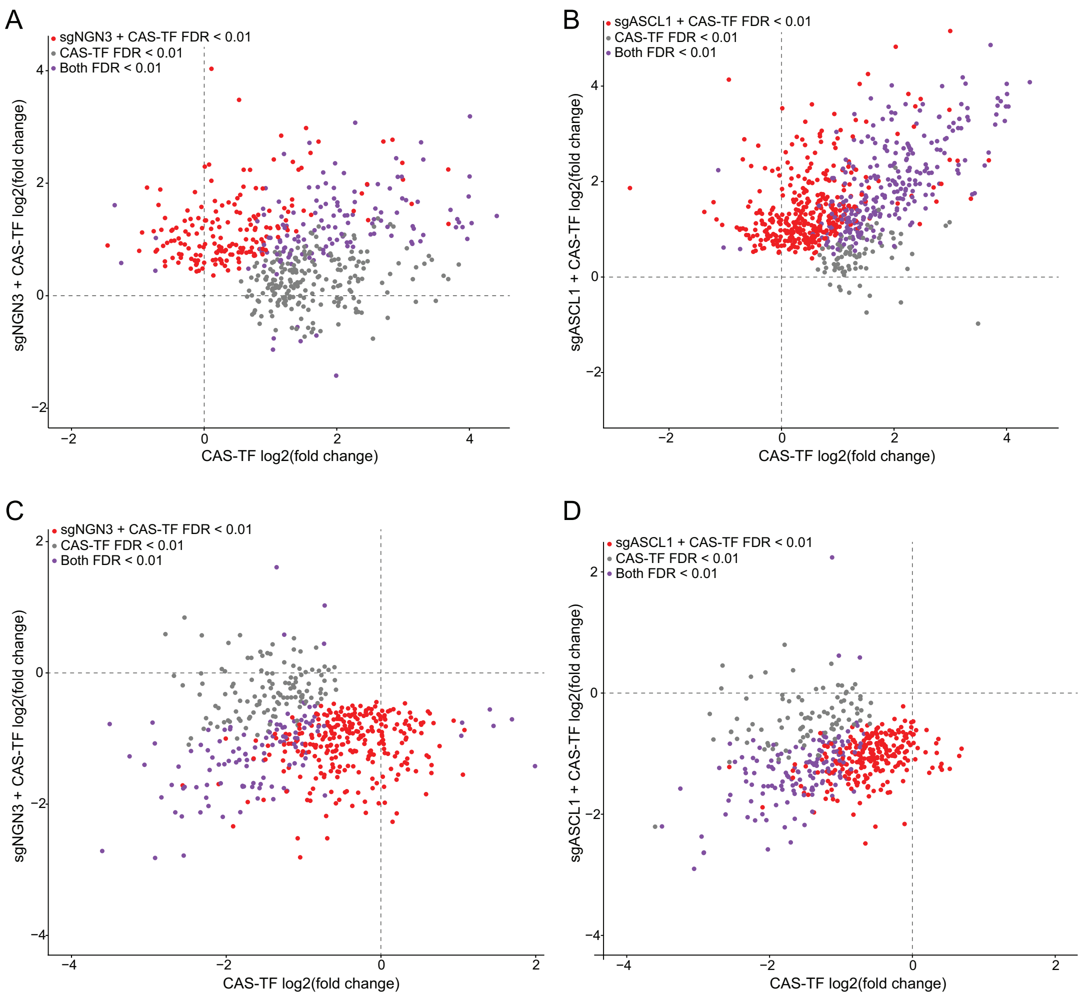

Figure S5. Comparisons of the single factor and paired CAS-TF screens. The fold change in gRNA abundance between mCherry-High and mCherry-Low expressing cells for the (A and B) sgNGN3 versus single factor CAS-TF screens for all positively (A) and negatively (B) enriched gRNAs across both screens and (C and D) sgASCL1 versus single factor CAS-TF screens for all positively (C) and negatively (D) enriched gRNAs across both screens. Related to Figure 3.

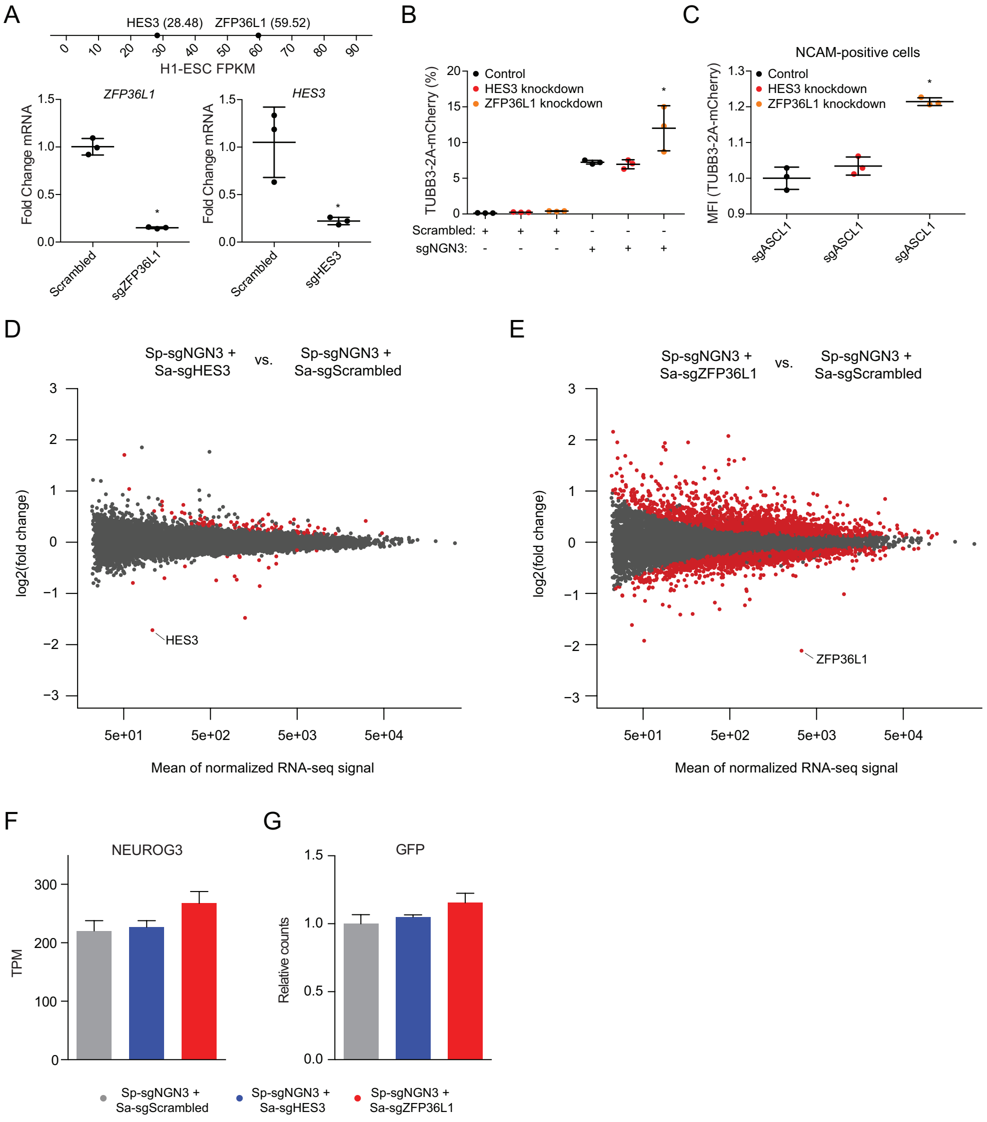

Figure S6. Gene activation and repression with orthogonal CRISPR systems. (A) Targeted repression of *ZFP36L1* and *HES3* in pluripotent stem cells using dSaCas9^{KRAB} targeting the promoter with a single gRNA for seven days (*p < 0.05 by two-tailed t-test, n = 3 biological replicates, error bars represent SEM). Effects on differentiation with either sgNGN3 (B) or sgASLC1 (C) in *ZFP36L1* and *HES3* knockdown cell lines (*p < 0.05 by global one-way ANOVA with Dunnett's post hoc test comparing all groups with either sgNGN3 or sgASCL1 to the Control cell line that received a scrambled non-targeting *S. aureus* gRNA, n = 3 biological replicates, error bars represent SEM). Differential expression analysis for sgNGN3-derived neurons with (D) *HES3* knockdown and (E) *ZFP36L1* knockdown. Red data points indicate FDR < 0.01 by differential expression analysis with DESeq2 (n = 3 biological replicates). (F) Expression of the *S. pyogenes* gRNA target gene, *NEUROG3,* across the three conditions shown. (G) GFP expression on the *S. pyogenes* gRNA lentiviral vector was used as a proxy for transduction level and gRNA expression across the three conditions shown. Related to Figure 6.

Table S1. All positive hits across the three neuronal differentiation screens. Related to Figures 1, 3 and 6.

Single Factor CAS-TF	sgNGN3 + CAS-TF	sgASCL1 + CAS-TF
NEUROG3	PRDM1	RUNX3
SOX4	LHX6	PRDM1
SOX9	NEUROG3	KLF6
KLF4	PAX8	PAX2
NR5A1	SOX3	RFX3
NEUROD1	KLF4	SOX10
SOX17	FLI1	GATA1
SMAD1	FOXH1	KLF5
ATOH1	FEV	KLF1
INSM1	SOX17	ERF
NEUROG1	FOS	LHX6
SOX18	INSM1	PHOX2B
RFX4	SOX2	NANOG
KLF7	WT1	NR5A2
SP8	SOX18	ETV3
OVOL1	ZNF670	NEUROG3
NEUROG2	LHX8	SOX4
ERF (from sublibrary)	OVOL1	SOX9
PRDM1 (from sublibrary)	E2F7	PAX8
OLIG3 (from sublibrary)	AFF1	IRF5
HIC1 (from sublibrary)	HMX2	CDX4
SOX3 (from sublibrary)	MAZ	RARA
FOXJ1 (from sublibrary)	RARA	BHLHE40
SOX10 (from sublibrary)	PROP1	SOX3
KLF6 (from sublibrary)	FOSL1	KLF4
ASCL1 (from sublibrary)	PAX5	NR5A1
PLAGL2 (from sublibrary)	KLF3	IRF4
		ASCL1
		GATA6
		SPIB
		THRB
		FOXH1
		NEUROD1
		SOX17
		CDX2
		ZEB2
		RARG
		INSM1
		FOSL1
		NEUROG1
		SOX1
		WT1

		PAX5
		SOX18
		POU5F1
		RFX4
		KLF7
		NKX2-2
		OVOL2
		FOXJ1
		PRDM14
		VENTX
		LHX8
		GFI1
		KLF17
		OVOL1
		OLIG3
		HMX3
		ZNF521
		ONECUT3
		OVOL3
		ZNF362
		AFF1
		HMX2
		ZNF786
		GATA5
		TBX3
		ZNF385A
		ATOH1
		PROP1
		SOX11
		JUN
		FOXE3
		FERD3L
		E2F7

Table S2. All negative hits across the three neuronal differentiation screens. Related to Figures 1, 3 and 6.

Single Factor CAS-TF	sgNGN3 + CAS-TF	sgASCL1 + CAS-TF
ZIC2	HES2	ETV1
SPI1	SREBF1	ZIC2
GRHL2	CIC	GSC2
TFAP2C	WHSC1	CIC
KLF8	VDR	GRHL2
MYB	HES1	REST
TCF21	ID2	TFAP2C
KLF12	TCF21	SALL1
TWIST1	SNAI1	NFKB1
SNAI1	RREB1	ELF2
RREB1	GCM2	HES1
GCM2	IRF3	MYB
GRHL1	FOXA1	KLF12
ETS1	GATA5	VSX2
BARHL2	GRHL1	NFE2
GRHL3	SOX5	SNAI1
ELF3	DMRT1	TRERF1
PTF1A	GCM1	RREB1
GSX1	BARHL2	IRF1
PBX2	SOX13	IRF3
NOTO	ZEB1	KLF2
KLF3	PITX2	MYOD1
ZNF311	PTF1A	SOX15
ELMSAN1	ZNF282	BARX1
ZNF296	NPAS2	GRHL1
PLEK	ZNF160	SOX5
KMT2A	HES7	ETS1
HES3	ZBED4	SKIL
	SALL4	BARHL2
	GLIS3	SOX13
	TBX22	ERG
	ZNF331	GRHL3
	EGR4	ZNF281
	ZIC5	ELF3
	ZNF710	HESX1
	ZNF697	KLF15
	ZFP36L2	PITX2
	ELMSAN1	PTF1A
	ZNF296	GSX1

	ZNF318	ZNF160
	ZNF570	ETV5
	ZNF683	MYBL1
	ZFP36L1	NOTO
	HES4	DPF1
	ZNF777	MECOM
	HES5	GLIS3
	ZIM2	KLF3
	ZNF579	TBX22
	BMP2	ESX1
	CRAMP1L	ZNF337
	TOX3	ZFP36L2
	FEZF2	ELMSAN1
	HES3	ZNF618
	ZNF791	ZNF296
		ZNF318
		ZNF570
		ZNF497
		ZFP36L1
		HES5
		BMP2
		CRAMP1L
		ZNF821
		KMT2A
		HES3
		BSX

Table S3. Protospacer sequences for all gRNAs used in this study. Related to STAR Methods.

Gene	sgRNA Sequence
Scrambled 1	TGTCGTGATGCGTAGACGG
Scrambled 2	TCATCAAGGAGCATTCCGT
NEUROG3	CTCGAGAGAGCAAACAGAG
RFX4	ATAGAAGGGGGAAGTCGGA
SOX4	CATGCCAAACCCCTCCCCC
NEUROD1	TGAGGGGAGCGGTTGTCGG
INSM1	CGCCGGGCGGGGCGACCAG
KLF7	AGCGCGAGCGCAAGGGACA
SOX9	CTGGGTGACGAGGCGGGAG
SOX17	CAAGGCTACACCTGCCCCC
NEUROG1	TAGCCCGAGCCGACTCCCG
SP8	GCGCGCGCCGTGAGGTCAT
KLF4	CTCCCTTCCATCGTTGCTA
SMAD1	CCGGGCCGGGAATTTGGAG
OVOL1	CGACAGGTAACAAATAGGT
NR5A1	AATACCCCTATCTATCTGG
ATOH1	GCCTGCCCGCGCCCTCCAT
NEUROG2-1	GCAGCGAGGACGAAGGCGG
NEUROG2-2	GGAAAGGCGGTGAAGAAAG
SOX18	GCCTCAGCGGAATCCCGCC
ASCL1	GAGGAGGAGGGGGAGTTTA
ASCL1 (sublibrary)	AATGGAGAGTTTGCAAGGAG
ATOH7	ACTAACACACCATCTGGAG
ATOH8	CGGGGCGGTTGTGCAGGAG
ATOH1-2	GGCTGAGAAGACACGCGAC
ATOH1-3	CACTCGGAGATCACACACC
ATOH1-4	CACGCGACCGGCGCGAGGA
ATOH1-5	TGCGGAGCCGGCTCTCGGC
NR5A1-2	AGAGAAACACCAACAAAGA
NR5A1-3	GGCCTGCAGAGTCACGTGG
NR5A1-4	TGCCCCCACGTGACTCTGC
NR5A1-5	GGGCCACCGGAGGCCCAAT
LHX6	AGGAGGAGGACTACCAAGA
LHX8	CGGGGAACACCGGGCTAAA
E2F7	GCGCCAAGACTCCGAGGGG
RUNX3	CCTGCCGGAGGCCGCCCAA
FOXH1	CCACCCAAAGGCAACTCAG
SOX2	GGATACAAAGGTTTCTCAG
HMX2	AGGCCCTCGGCGCGCTCTG
NKX2.2	CCCTCTAGAGCAAGATGAG
ELMSAN1	GGCGTCCTTAAACCTCAGG
GCM2	ACAGTCCCAGGAACGGAGG

HES1	GTGGACCGCGCCCCCCCAT
HES7	CCCTCTAGGACCCGGCACG
TOX3	AGAAGAGGGGCCCCGGAGA
DMRT1	GGACCCTGCAGCAAAGCCC
BMP2	CCGCCCGCTCGGGGATCCC
ZFP36L1	CTTCCCTACCCGGCGCTTC
ERF	GAGCGTGTGTGTGAGTGCGC
PRDM1	CGGCTGTGCTAGCAATCTGG
OLIG3	GAGCCCTCCTATCTATCCT
HIC1	GCTGTGCGCCGTGCCCGCCC
SOX3	CGGAGGACCCGTGATTGAC
FOXJ1	GCTCGGCTCATTCCCGCCCG
SOX10	CCCTGAGTGTTGGGGATGA
KLF6	TCCCGTGGCTCCCGGCCCGG
PLAGL2	GCCCCGGCCGCTCTAGCCCG
S. aureus Scrambled	TCATCAAGGAGCATTCCGT
S. aureus ZFP36L1	ATGACAACAAGAACCCCGGA
S. aureus HES3	CCCTTCCCCGGGAGGTGTGG

Table S4. All qRT-PCR primers used in this study. Related to STAR Methods.

Gene	Primer Sequence
NCAM Fwd	AACCCAGTGCACCTAAGCTC
NCAM Rev	GGACTTCAGCATGACGTGGT
MAP2 Fwd	CAGCTTGTCTCTAACCGAGGA
MAP2 Rev	TGTGTCGTGTTCTCAAAGGGT
TUBB3 Fwd	TTTGGACATCTCTTCAGGCC
TUBB3 Rev	TTTCACACTCCTTCCGCAC
ZFP36L1 Fwd	CCGAGTCCCCTCACATGTTT
ZFP36L1 Rev	TTGAGTTGTCCAAGGTCGGG
HES3 Fwd	GAAAGTCTCCCTGGCTCGTC
HES3 Rev	CCAAATAGGGAGCGCCTTCA
NEUROG3 Fwd	TTTTCTCCTTTGGGGCTGGG
NEUROG3 Rev	AGGCGTCATCCTTTCTACCG
RFX4 Fwd	GACGAGCGGCCATTCATCAG
RFX4 Rev	CACTCAGTAATCCAGCCGGG
SOX4 Fwd	AACAGGGCGGCTGGTTAATA
SOX4 Rev	ACACTGGTGGCAGGTTAAGG
NEUROD1 Fwd	GATGACTAAGGCTCGCCTGG
NEUROD1 Rev	AGAATAGCAAGGCACCACCT
INSM1 Fwd	TACGCGTTTGTCTCGTGGTT
INSM1 Rev	CAGAGATTGGTAGGCGAGGC
KLF7 Fwd	TTGCATTAGGAGCGAACAGC
KLF7 Rev	AAAAGGGGACTTCTCCACGG
SOX9 Fwd	TAAAACGGTGCTGCTGGGAA
SOX9 Rev	AGTGTGCTCGGGCACTTATT
SOX17 Fwd	GACATGAAGGTGAAGGGCGA
SOX17 Rev	CGTTGTGCAGGTCTGGATTC
NEUROG1 Fwd	AATATCTCCCGGGCGTCTGA
NEUROG1 Rev	GTTCAAGTTGTGCATGCGGT
SP8 Fwd	CTTCTAGGGGAAGAACCGAGG
SP8 Rev	AAGAGGACGAGGAGCGTTTC
KLF4 Fwd	CACCGGACCTACTTACTCGC
KLF4 Rev	AACCCCAAATTGGCCGAGAT
SMAD1 Fwd	GGAGAAAGGAGAGGCCGAGC
SMAD1 Rev	AAAAGTAACCCAGTCAGCACCG
OVOL1 Fwd	GTCCGGCTCGCACTTTAAGA
OVOL1 Rev	CTGAGAACGAGGTCCCTTGC
NR5A1 Fwd	GTGGTGTGAGGGGGTTTCTG
NR5A1 Rev	TACGAATAGTCCATGCCCGC
ATOH1 Fwd	AGGATGCATGGGCTGAACC
ATOH1 Rev	TTGTAGCAGCTCGGACAAGG
NEUROG2 Fwd	CAGGCCAAAGTCACAGCAAC
NEUROG2 Rev	CGATCCGAGCAGCACTAACA

SOX18 Fwd	GCAAAGGACGAGCGCAAG
SOX18 Rev	CTTGTAGTTGGGGTGGTCGC
SOX11 Fwd	AGCGGAGGAGGTTTTCAGTG
SOX11 Rev	TTCCATTCGGTCTCGCCAAA
